# Targeting the epigenome with advanced delivery strategies for epigenetic modulators

**DOI:** 10.1002/btm2.10710

**Published:** 2024-08-17

**Authors:** Sonia Guha, Yogeswaran Jagadeesan, Murali Monohar Pandey, Anupama Mittal, Deepak Chitkara

**Affiliations:** ^1^ Department of Pharmacy Birla Institute of Technology and Science Pilani (BITS Pilani) Jhunjhunu Rajasthan India

**Keywords:** biomacromolecules, CRISPR/Cas tool, epigenetic modulators, epigenetics, nanocarriers, prodrugs

## Abstract

Epigenetics mechanisms play a significant role in human diseases by altering DNA methylation status, chromatin structure, and/or modifying histone proteins. By modulating the epigenetic status, the expression of genes can be regulated without any change in the DNA sequence itself. Epigenetic drugs exhibit promising therapeutic efficacy against several epigenetically originated diseases including several cancers, neurodegenerative diseases, metabolic disorders, cardiovascular disorders, and so forth. Currently, a considerable amount of research is focused on discovering new drug molecules to combat the existing research gap in epigenetic drug therapy. A novel and efficient delivery system can be established as a promising approach to overcome the drawbacks associated with the current epigenetic modulators. Therefore, formulating the existing epigenetic drugs with distinct encapsulation strategies in nanocarriers, including solid lipid nanoparticles, nanogels, bio‐engineered nanocarriers, liposomes, surface modified nanoparticles, and polymer–drug conjugates have been examined for therapeutic efficacy. Nonetheless, several epigenetic modulators are untouched for their therapeutic potential through different delivery strategies. This review provides a comprehensive up to date discussion on the research findings of various epigenetics mechanism, epigenetic modulators, and delivery strategies utilized to improve their therapeutic outcome. Furthermore, this review also highlights the recently emerged CRISPR tool for epigenome editing.

**Abbreviations**
AEs: artificial exosomes
AML: acute myeloid leukemia
CpG: 5′‐cytosine‐phosphate‐guanine‐3′
CRISPR/Cas: clustered regularly interspaced palindromic repeats/CRISPR‐associated protein
EE: epi‐effectors
EM: epigenetic modifiers
EM: epigenetic modulators
GBM: glioblastoma multiforme
HSANPs: HSA nanoparticles
hTERT: human telomerase reverse transcriptase
miRNA: microRNA
ncRNA: noncoding RNA
NIR: near‐infrared range
nt: nucleotides
PAMAM: poly(amidoamine)
PcG: polycomb group proteins
PD: Parkinson's disease
PEG: polyethylene glycol
PEI: polyethyleneimine
piRNAs: Piwi‐interacting RNAs
sgRNA: single guide RNA
siRNA: small interfering RNA
TM: transcription modifiers

**Translational Impact Statement**

Utilizing advanced delivery strategies such as nanoformulations for the delivery of epigenetic modulators holds immense translational promise, providing a sophisticated platform for precise and targeted manipulation of gene expression. This innovative approach not only enhances the efficacy of therapeutic interventions but also minimizes off‐target effects, thereby advancing the frontier of clinical precision medicine and broadening the scope of tailored therapeutic approaches for diverse conditions, spanning from oncological malignancies to neurological disorders, ultimately revolutionizing disease management through targeted epigenetic interventions.

## 1 INTRODUCTION

**FIGURE 1:**
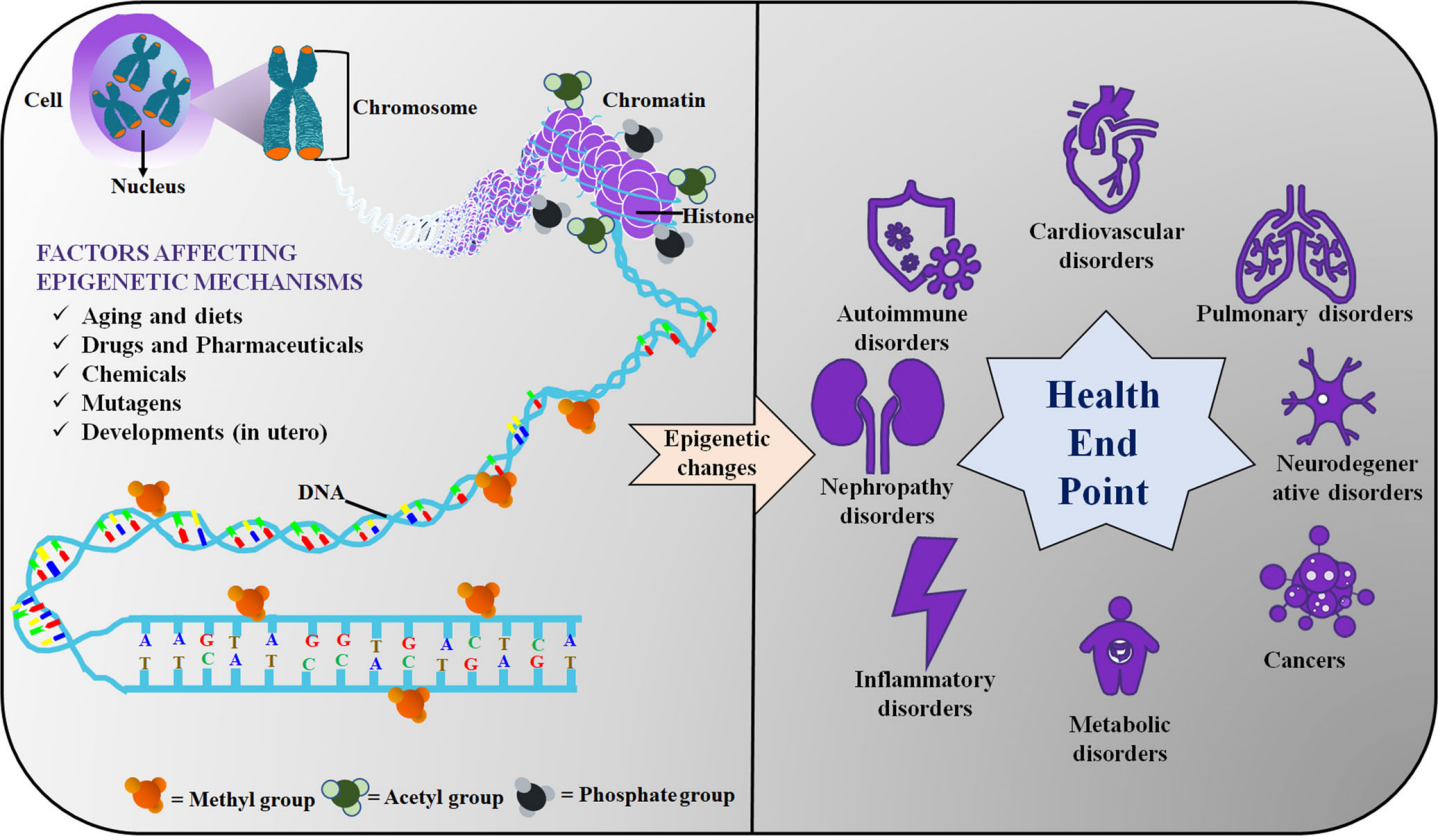
Factors affecting epigenetic mechanisms and complications associated with epigenetic alterations.

Epigenetics, meant as “beyond” genetics, was coined by a British biologist, Conrad H. Waddington, referring to the phenotypic changes not coded by DNA.^1^ The occurrence of disorders and diseases may be possible even when there is no change/mutation in genetic material. It has been shown that different epigenetic mechanisms regulate the arrangement of DNA structure resulting in aberrant gene expression that may lead to several diseases and disorders.^2^, ^3^, ^4^ Now it is well established that epigenetic mechanisms are significant in the initiation and development of different diseases, such as cancers, multiple sclerosis, asthma, metabolic, cardiovascular and neurological disorders, and so forth (Figure 1).^5^, ^6^, ^7^, ^8^ Although different epigenetic mechanisms are involved in development of cancers, methylation status of 5′‐cytosine‐phosphate‐guanine‐3′ (CpG) islands in the promoter site of the tumor suppressor gene (e.g., retinoblastoma, breast cancer, ovarian cancer) have been studied extensively. Modification of histone protein and CpG moieties can also promote cardiovascular diseases and atherosclerosis as well.^9^, ^10^ For instance, a study conducted by Post et al. exhibited that human atheroprotective estrogen receptors genes such as ESR1 and ESR2 were found to be frequently hypermethylated in the case of atherosclerosis.^11^ Similarly, a well‐known progressive neurodegenerative disorder, Huntington's disease is instigated by an extended polyglutamine repeat sequence in huntingtin protein, which can enter the nucleus and able to bind as well as inhibit histone acetyltransferases (HATs). Hence, decreased histone acetylation of H3 and H4 leads to neuronal gene silencing.^12^, ^13^, ^14^ Likewise, different human diseases are originated due to alteration of different epigenetic statuses including DNA methylation, histone modifications including methylation, acetylation, deacetylation, ubiquitination, and phosphorylation. Subsequently, researchers have paid much attention to inhibit reversible changes in epigenetic status by epigenetic modulators, which can have significant therapeutic potential.

Desirable improvement in disease treatment through epigenetics could not only be accomplished by discovering novel drugs and finding new targets but also through an efficient delivery strategy that provide a site‐specific delivery to achieve the required therapeutic concentration. Further, as the epigenome is a complex and highly regulated process, delivery of epigenetic modulators through a suitable delivery system is needed to avoid the off‐target effects along with improvement in therapeutic effects. Periodically, treatment with the existing drug therapy remains challenging due to increased drug resistance, toxicity, nonspecific target, and poor bioavailability. To overcome these hindrances, advanced delivery strategies are much needed. Thus, nanotechnology becomes the impeccable option for drug delivery as it enhances the bioavailability and drug efficacy, reduces drug dose, administration frequency, and toxicity, and leads to improved pharmacokinetic profile. Many reports are available on the utilization of nanocarriers, such as liposomes, polymer–drug conjugates, nanogel, solid lipid nanoparticles, exosomes, and surface modified nanomaterials for an effective delivery system for epigenetics drugs.^15^, ^16^, ^17^, ^18^, ^19^, ^20^, ^21^ Herein, we highlight different delivery system for epigenetic therapy and their mechanism, different epigenetic modulators as sole drug, structure‐altered drug, prodrug, combination therapies, and biomacromolecules for enhanced therapeutic efficacy.

## 2 EPIGENETICS: AN OVERVIEW

At the very beginning, epigenetics is called as “epigenesis”, a word derived from Greek meaning “above genes”. However, the word epigenetics has been used in an ambiguous manner in the past. Later, “epigenesis” was transformed into “epigenetics”, the domain which deals with understanding of genes and its regulation, heredity, modern biology and medicine.^22^ More precisely, epigenetics is defined as heritable, somatic, and stable changes in gene expression, mainly associated with chromosomal alteration instead of changes in DNA sequence. Although the epigenetic mechanisms are not directly involved in the alteration of DNA sequences, chemical modifications of DNA bases along with alteration of the superstructure of chromosomes are responsible for the regulation of gene expression by epigenetic mechanism.^23^ The nucleosome, a complex of positively charged histone protein octamer which comprises two units of each H2A, H2B, H3, and H4, is tightly packed around 146 base pairs or 1.7 times of negatively charged DNA. This complexed nucleosome is a chromatin fiber's primary unit which is further condensed to form chromosomes.^24^, ^25^, ^26^ The chromatin fibers exist in two states: (1) euchromatin (loose and transcriptionally active form) and (2) heterochromatin (dense and transcriptionally inactive state). Intermittently, due to chemical modifications, the structural form of chromatin can be shifted to active euchromatin and inactive heterochromatin, which govern the gene expression and suppression, respectively.^3^ Besides histone modification, DNA methylation also plays a significant role in epigenetics by allowing methylation in CpG islands of promoter sequences resulting in gene expression silencing.^27^ In addition, noncoding RNA (ncRNA) sequences are also necessary for regulating gene expression through epigenetic mechanisms.^28^

## 3 EPIGENETIC MECHANISMS

**FIGURE 2:**
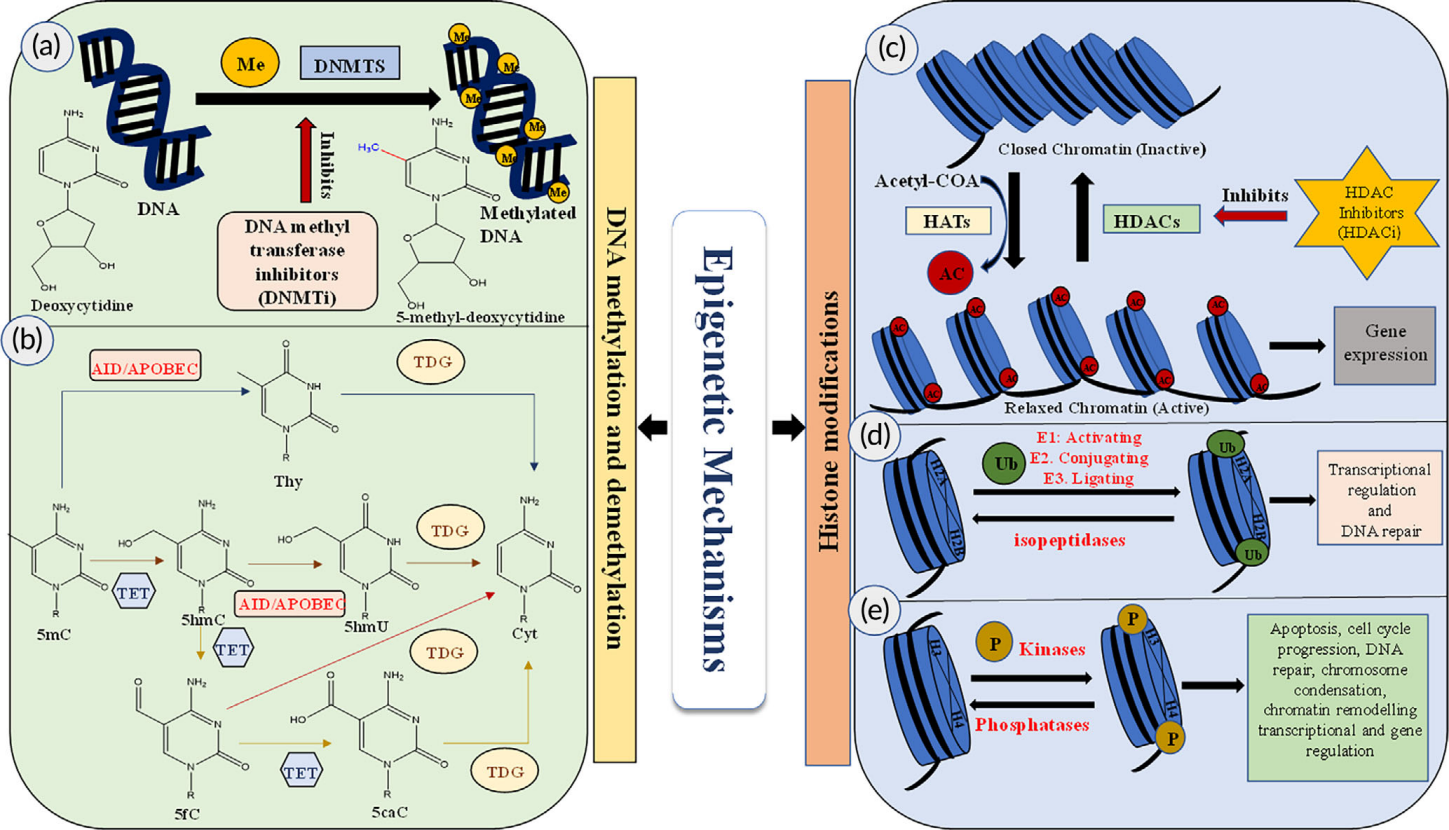
Schematic diagram of different epigenetic mechanisms. (a) DNA methylation. (b) Pathway of DNA demethylation. (c) Histone acetylation and deacetylation. (d) Histone ubiquitination. (e) Histone phosphorylation. 5caC, 5‐carboxyl cytosine; 5fC, 5‐formyl cytosine; 5hmC, 5‐hydroxymethylcytosine; 5hmU, 5‐hydroxymethyluracil; 5mC, 5‐methylcytosine; Ac, acetyl group (–COCH_3_); AID, activation‐induced deaminase; APOBEC, apolipoprotein B mRNA editing enzyme, catalytic polypeptide; Cyt, cytosine; DNMTs, DNA methyltransferases; HATs, histone acetyltransferases; HDACi, histone deacetylase inhibitors; HDACs, histone deacetyl transferases; Me, methyl group (–CH_3_); P, phosphate group; TDG, thymine DNA glycosylase; TET, Ten‐eleven translocation enzymes; Thy, thymine; Ub, ubiquitin.

Recently, several studies were performed to understand the epigenetic mechanisms and their role in different human diseases. Epigenetic mechanisms are broadly classified into three different classes based on their mode of action, such as DNA methylation, histone modification, and ncRNA (Figure 2). All these epigenetic systems mainly focussed on regulating gene expression by modulating chromatin structure and allowing the transcription factors to bind at the promoters site and transcription elongation site except microRNA (miRNA) and small interfering RNA (siRNA), which commonly come under the classification of ncRNA.

### 3.1 DNA methylation

DNA methylation is well known for its gene silencing among all the epigenetic mechanisms. In recent years, DNA methylation has been widely used in different cell biology studies, including inactivation of chromosome X, embryonic development, genomic imprinting, and gene silencing or overexpression at different evolution stages. DNA methylation is a process of adding a methyl group (–CH_3_) at the fifth carbon (C^5^) of the cytosine ring found in the CpG dinucleotide site to form 5‐methylcytosine (5mC) with the help of the enzyme DNA methyltransferase (DNMTs; Figure 2a).^29^ CpG island comprises many repetitive sequences of CpG dinucleotides in the promotor region of genes in the somatic cells. DNA methyltransferases (DNMTs) such as DNMT1, DNMT2, DNMT3A, DNMT3B, and DNMT3L are primarily involved in DNA methylation using a methyl donor named S‐adenosyl‐methionine.^30^ DNA methylation‐mediated gene silencing mechanism either obstructs the binding of transcription factors or regulatory components with the promotor region of DNA or binding methyl‐binding proteins (MBPs) such as MBD1, MBD2, MBD3, and MBD4, which leads to gene suppression by interacting with a co‐repressor complex.^31^, ^32^ However, hypomethylation at a particular region switches on the gene expression. Hypomethylation causes genetic mutations in DNA sequences resulting in genetic instability, whereas reports have evidenced that methylation showed genome stability.^33^

Diseases that evolved due to DNA methylation include tumorigenesis, lupus, muscular dystrophy, autoimmune diseases, metabolic and psychological disorders, aging, and a series of congenital abnormalities.^29^, ^34^ Since the mechanism of demethylation is not well known, recent reports revealed that the demethylation process is more complex and follows both active and passive pathways (Figure 2b). Active demethylation occurs either through the deamination process of 5mC by activation‐induced deaminase (AID), APOBEC‐family cytosine deaminases (apolipoprotein B mRNA editing enzyme, catalytic polypeptide), or Ten‐eleven translocation (TET) enzymes which assist in oxidization process of 5mC and removal of locus‐specific DNA methylation.^35^, ^36^, ^37^, ^38^, ^39^ Notably, water, oxygen, and α‐ketoglutarate promote the conversion of 5mC to 5‐hydroxymethylcytosine (5hmC), 5‐formyl cytosine (5fC), and 5‐carboxyl cytosine (5caC) by the bit‐by‐bit oxidation process, alongside the formation of carbon dioxide and succinate.^37^ Thereafter, the replacement of 5fC and 5caC with unmodified cytosine is catalyzed by the base‐excision repair enzyme, thymine DNA glycosylase (TDG). Other suggested mechanisms of DNA demethylation are less explored, including decarboxylation of 5caC, removal of the hydroxymethyl group of 5hmC by DNMT, and deamination of 5hmC and 5mC by the AID and APOBEC.^36^

### 3.2 Histone modification

Histones are small, highly alkaline proteins found in chromatin structures that play a crucial role in gene regulation through epigenetic mechanisms. From earlier understanding, histone is mainly known as a DNA packaging material without contributing to gene regulation. However, histones have been demonstrated to undergo post‐translational modifications, including methylation, acetylation, deacetylation, phosphorylation, ubiquitination, and SUMOylation (small ubiquitin‐like modifier; Figure 2c–e).^3^ Among the post‐translational modifications, acetylation and methylation are the most extensively studied and reported in the literature. Histone acetylation is a dynamic process regulated by two families of enzymes such as histone acetyltransferase (HAT) and histone deacetylase (HDAC).^40^, ^41^ Histone acetylation occurs at lysine (Lys) residues in the tails of histone proteins H2B, H3, and H4. This specific modification is mediated by HATs, which promote the shift of an acetyl group from acetyl‐CoA to the lysine ϵ‐amino groups at the N‐terminal tails of histones.

In contrast, HDAC exactly counteracts this activity. In addition, the acetylation of histones weakens the electrostatic interactions between histone protein and DNA, becoming relaxed chromatin that upregulates transcription due to increased access to transcription factors and RNA polymerase II.^42^ Hence, histone acetylation enhances gene expression. HATs are classified into types A and B, in which type A includes Gcn5‐related N‐acetyltransferases, MYST (MOZ, Ybf2, Sas2, and Tip60), and p300/CBP families of enzymes that are found in the nucleus. Type B HATs are located in the cytosol and promote the acetylation of free histones or non‐histone proteins. Whereas HDACs embodies with four different classes such as class 1 (HDAC1, 2, 3, and 8), class 2 (2a: HDAC4, 5, 7, 9; 2b: HDAC 6, 10), class 3 (SIRT), and class 4 (HDAC11) which repress gene expression by condensing nucleosomes due to removal of acetyl groups from acetylated proteins.^43^ Moreover, HATs and HDACs are involved in their own target regions with specific transcriptional factors.^44^ Over the last decade, histone methylation has become an essential epigenetic tool in regulating gene expression. Histone methylation can either activate or repress the transcription based on the degree of methylation, such as mono‐, di‐, and tri‐methylation on the particular methylated Lys or Arginine (Arg) residue and the position of methylation.^45^ For example, histone methylation of H3K4, H3K36, and H3K79 causes transcription of genes, whereas methylation of H3K9, H3K27, and H4K20 results in silencing of transcription.^3^, ^46^ Furthermore, the mono‐methylation of Lys at the H3K9 position results in transcription activation, and tri‐methylation at the same position inactivates transcription. The regulation of histone methylation is very dynamic and catalyzed by two types of enzymes, including histone methyltransferases (HMTs) and histone demethylases (HDMTs).^47^ Briefly, lysine methyl transferase (KMT) and protein arginine methyl transferase (PRMT) are involved in histone methylation on Lys (K) and Arg (R) residues, respectively. Later, lysine‐1 specific demethylase enzyme (LSD1), which is also called KDM1A, was reported for histone demethylase activity on H3 Lys 4 (H3K4).^48^

In addition to this, histone phosphorylation plays a significant role in apoptosis, cell cycle progression, gene regulation, DNA repair, chromatin remodeling transcriptional and chromosome condensation.^49^, ^50^, ^51^, ^52^ Histone phosphorylation is a reversible process that takes place on serine (Ser), threonine (Thr), and tyrosine (Tyr) residues by kinases and phosphatases through either adding or removing phosphate moiety resulting in condensation of chromatin during cell division.^53^ During mitosis cell division, phosphorylation of Thr119 located in H2A is involved in regulating chromatin function as well as structure, and phosphorylation of Ser10 located in H3 (H3S10) is linked to chromatin compaction. In addition, phosphorylation of H3S10 was also reported for transcriptional activation.^3^, ^54^ Furthermore, phosphorylation of H2A(X) at Ser139 (γ‐H2AX) leads to DNA repair.^55^, ^56^

Ubiquitination, a type of histone modification, was first found in Histone H2A.^57^, ^58^ Besides ubiquitinated H2A (uH2A), ubiquitinated H2B (uH2B) has also been reported. Although, uH2B is less abundant (1%–2%) as compared to uH2A (5%–15%).^59^ Mostly, a monoubiquitinated form of H2A (uH2A) and H2B (uH2B) has been reported; however, the polyubiquitination form of H2A was only detected.^60^ Ubiquitination occurs by adding a ubiquitin moiety in Lys residues such as Lys 119 and 120 located at H2A and H2B, respectively, and regulated by a sequential action of three different enzymes such as activating (E1), conjugating (E2), and ligating (E3). On the other hand, isopeptidases are involved in the de‐ubiquitination process by removing the ubiquitin moiety.^61^, ^62^, ^63^

In 2003, histone SUMOylation was demonstrated for the first time by Shiio et al., when it was observed in H4 of human histone protein.^64^ SUMO proteins can be covalently attached to the Lys residue of substrate proteins mediated by E1, E2, and E3 enzymes, similar to the ubiquitylation pathway.^65^ SUMOylation of histone regulates different cellular processes such as transcription, DNA repair, DNA replication, cell‐cycle progression, gene expression, apoptosis, mitochondrial dynamics, stress responses, and ribosome biogenesis.^66^, ^67^ De‐SUMOylation (SUMO deconjugation) is catalyzed by cysteine proteases known as SUMO‐specific proteases or SUMO isopeptidases.^68^

### 3.3 Noncoding RNAs

Noncoding RNAs (ncRNAs), which are not translated into proteins but transcribed from DNA, can be divided into two categories depending on size: long noncoding RNA (lncRNAs) and short chain noncoding RNAs, including short (or small) interfering RNAs (siRNAs), microRNAs (miRNAs), and Piwi‐interacting RNAs (piRNAs).^69^, ^70^ In recent years, ncRNAs have been considered essential epigenetic players as these interact with histone modifiers and DNA methyltransferases to regulate gene expression. LncRNAs greater than 200 nucleotides (nt) in length are located in the cytoplasm or nucleus and regulate the different genetic and epigenetic mechanisms of cellular functions, including maintenance of chromatin dynamics, DNA methylation, and changes in cell metabolism.^71^, ^72^ On the other hand, the mechanism of siRNA involves in post‐transcriptional gene silencing where siRNA specifically promotes the knockdown of mRNA of disease‐related genes efficiently. In the cytoplasm, siRNA binds to RNA‐induced silencing complex (RISC) and interacts with its Argonaute 2 component which results in unwinding of duplex structure of siRNA followed by degradation of the passenger strand of siRNA. The guide strand of siRNA is complementary to the mRNA of the targeted gene. Thereby, RISC complex is guided to the target mRNA by a guide strand of siRNA and induces mRNA degradation.^73^, ^74^, ^75^ Recent studies exhibited that siRNA can mediate transcriptional gene silencing (TGS) in cells through either DNA methylation or histone modification.^76^, ^77^, ^78^ Like siRNA, miRNA also shows a similar mechanism of action for TGS. The size of both identical fragments is approximately 19–24 nt in length.^79^, ^80^ miRNAs are mainly involved in changing the state of DNA or chromatin by inhibiting the activity of chromatin remodeling enzymes.^81^, ^82^ In addition, Lewis et al. demonstrated that histone deacetylases, histone methyltransferases, methyl CpG binding proteins, and chromatin domain proteins are potential targets for miRNAs.^83^ piRNAs associated with the Piwi protein also showed an inevitable role in the epigenetic mechanism by attaching with polycomb group proteins (PcG) and PcG response elements.^84^, ^85^, ^86^

## 4 EPIGENETIC MODULATORS AND THEIR DELIVERY STRATEGIES

**FIGURE 3:**
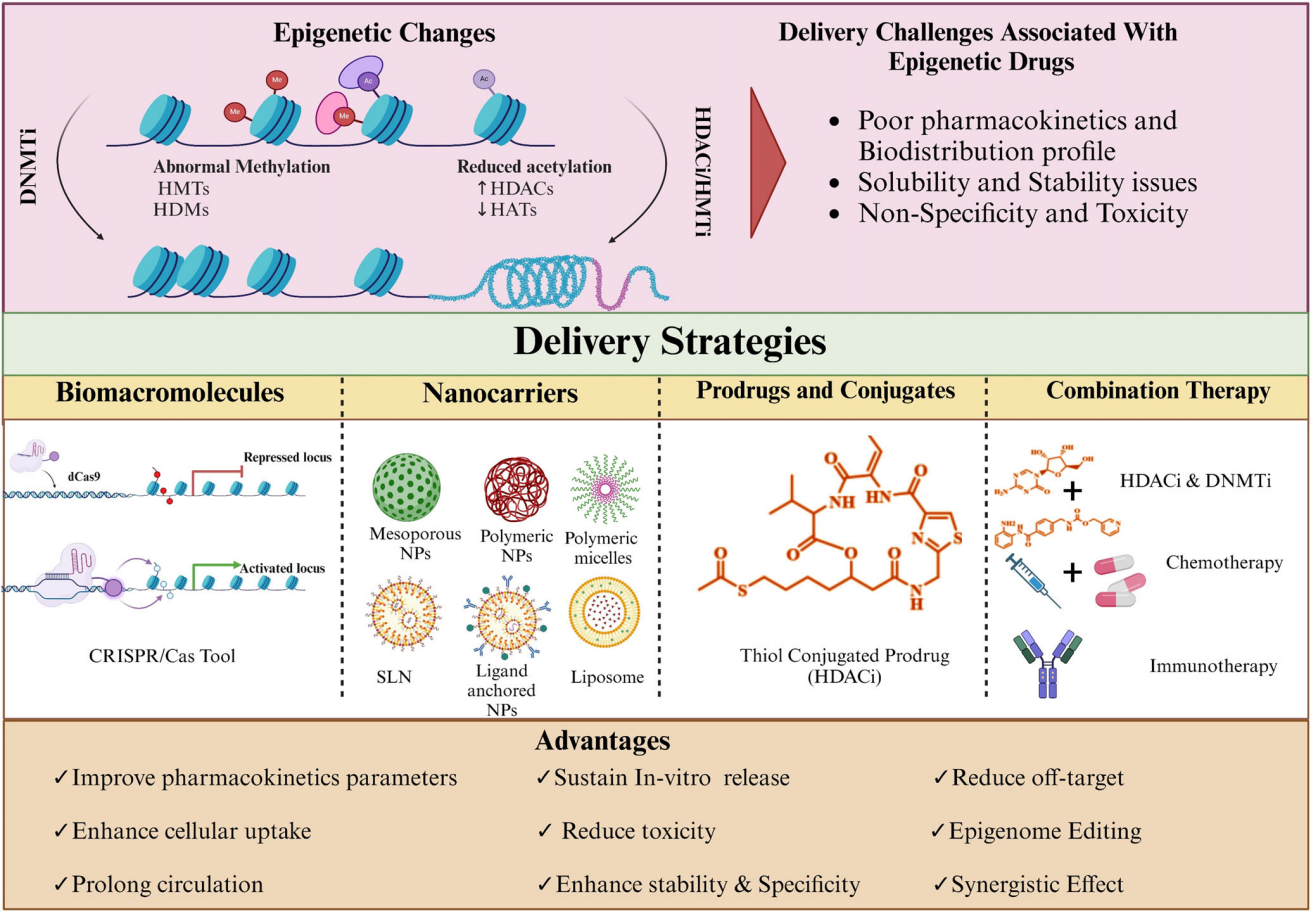
Different delivery strategies for epigenetic modulators.[Created with Biorender].

In recent years, numerous studies have focused on epigenetic drugs and their mechanism of action for various diseases. Additionally, research into delivery strategies for different epigenetic modulators has gained significant interest, aiming to enhance therapeutic outcomes and reduce side effects (Figure 3). Effective delivery methods for these epigenetic drugs are often necessary due to their rapid degradation by enzymes in vivo. For example, DAC is quickly degraded by cytidine deaminase, an enzyme present in high concentrations in tissues, such as the liver and gut. Furthermore, since DAC does not bind to proteins, it is excreted rapidly, necessitating continuous administration as an IV infusion.^87^, ^88^ On the other hand, targeted delivery can enhance drug concentration at the disease site, reduce systemic side effects, and improve patient compliance by potentially reducing the frequency of administration. In their efforts to target a wide range of diseases, researchers have developed various drug delivery systems, including polymeric and lipid‐based nanoparticles, polymer–drug conjugates, prodrug approaches, dendrimer, and micelle‐based nanocarrier, surface‐modified and bioengineered nanocarriers.^15^, ^16^, ^17^, ^18^, ^20^, ^21^ These specially designed delivery systems enhance the drug's selectivity for the target site, thereby increasing its efficacy. For instance, nanoscale delivery cargoes could enhance the therapeutic effectiveness of epigenetic drugs by protecting them from degradation, improving specificity and permeability to targets, and minimizing side effects. Surface modifications, including the addition of polyethylene glycol (PEG) or targeting ligands, could be engineered onto nanoparticles to enhance cellular uptake, extend blood circulation half‐life, and influence biodistribution.^89^ Several reports demonstrated the enhanced efficacy of epigenetic drugs when delivered via polymeric nanoparticles.^90^, ^91^, ^92^ Another strategy, micelle‐based nanocarriers have been also reported for epigenetic drugs. Polymeric micelles, formed through the self‐assembly of amphiphilic macromolecules, enable the oral delivery of poorly water‐soluble drugs. Enhanced oral bioavailability is achieved through the solubilization of lipophilic moieties in these micelles. Research groups have investigated the delivery of HDACi (e.g., Vorinostat and Panobinostat) using a micellar‐based delivery strategy that showed improved therapeutic efficacy as compared to free drug.^93^, ^94^

**FIGURE 4:**
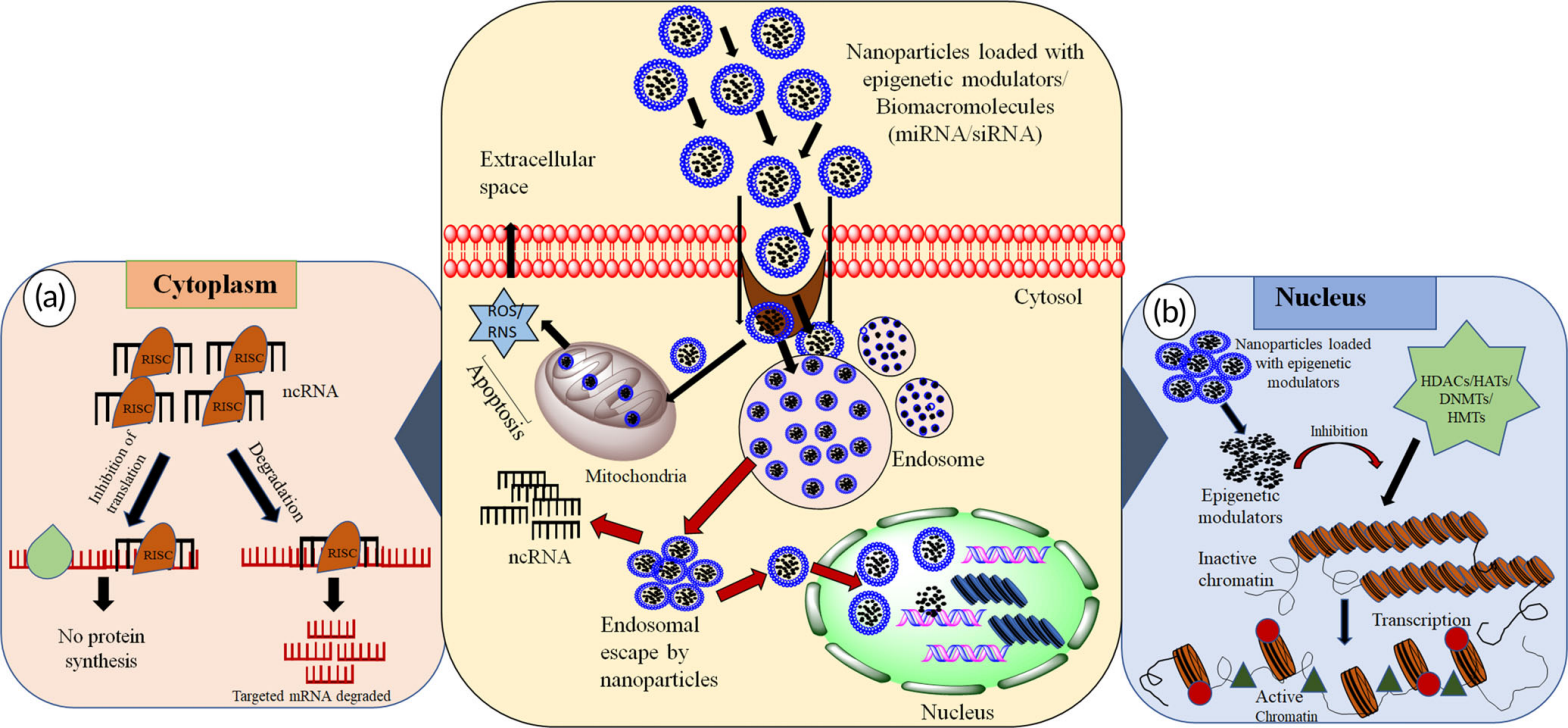
Intracellular fate of nanoparticles loaded with epigenetic modulators and noncoding RNA (ncRNA) in (a) cytoplasm and (b) nucleus.

In addition to polymeric nanoparticles and polymeric micelles, lipid‐based delivery strategies such as solid lipid nanoparticles, nanostructured lipid carriers, and liposomes have attracted researchers' attention as vehicles for epigenetic drugs. Lipid nanocarriers have been demonstrated to enhance intestinal permeability, protect drugs from acidic degradation, and improve oral absorption. For example, promising data supports the use of nanostructured lipid carriers for the oral delivery of decitabine.^95^ Moreover, both DNMTi and histone modifiers have been extensively explored using lipid‐based nanocarriers. In addition, dendrimers could enhance oral medication absorption by acting as permeability enhancers, altering intestinal epithelium barriers, and facilitating drug transport via paracellular and transcellular pathways. Conjugating dendrimers with PEG improves drug stability and solubility. Additionally, folate‐based targeting of tumors has demonstrated effectiveness, exemplified by the advancement of folate‐based dendrimer conjugates. Likewise, Zong et al. described the synthesis and biological evaluation of tumor‐specific dendrimer‐HDACi conjugates containing SAHA and folate units, highlighting their potential in targeted cancer therapy.^96^ On the other hand, prodrugs are biodegradable derivatives of active pharmaceutical components that remain inactive until they undergo enzymatic or chemical transformations to become active inside biological systems. Epigenetic drugs can be modified to enhance stability, slowing degradation in vivo, and improving targeting to specific delivery sites, either intracellularly or extracellularly. This is achieved by methods such as converting the parent drug into an ester, and exploiting varying in vivo esterase expression to cleave the prodrug and activate the drug. pH manipulation can also be employed to activate the drug specifically in the acidic conditions of tumor tissue.^97^ Similarly, a dinucleotide containing DAC, S110 showed reduced depletion by cytidine deaminase and similar efficacy to DAC in inhibiting DNA methylation. Alongside this, researchers are also investigating chemical modifications for existing epigenetic drugs.^98^ Likewise, CP‐4200, an elaidic acid derivative of azacytidine, demonstrated superior therapeutic efficacy in mouse models attributed to its chemical modification, which reduced dependence on nucleoside transporters.^99^ The following section summarizes reported studies on the delivery of drugs targeting epigenetic mechanisms through suitable cargoes and Figure 4 illustrates the intracellular fate of nanoparticles loaded with ncRNA and epigenetic modulators in cytoplasm (Figure 4a) and nucleus (Figure 4b).

### 4.1 DNA methyl transferase inhibitors

DNA methyl transferase inhibitors (DNMTi) are classified based on their chemical structure, such as nucleoside DNMTi, including Azacitidine, Decitabine, Zebularine, and so forth, and non‐nucleoside DNMTi, which can be either from a natural or synthetic source, small molecules, and antisense oligonucleotide.^100^ Nucleosidic DNMTi is known as the oldest form of DNA methylation inhibitors in which a few compounds like azacytidine (AZA) or 5‐Azacytidine (5‐Aza‐CR) (brand name: Vidaza®, Celgene, Summit, NJ) and decitabine (DAC) or 5‐aza‐2′‐deoxycytidine (5‐Aza‐CdR) (brand name: Dacogen®, MGI Pharma, Bloomington, MN) have been approved by US Food and Drug Administration (FDA) for the treatment of cancer.^101^ The nucleoside analog of DNMTi inhibits DNA methylation after incorporation into DNA during DNA replication. Further, it forms an irreversible covalent linkage with the DNMT enzyme that results in the degradation of the enzyme; hence, DNMT cannot promote DNA methylation.^102^ This may cause cell death and DNA damage due to obstruction of DNA synthesis and structural instability.^103^, ^104^, ^105^, ^106^ Besides nucleoside analogs, several non‐nucleoside DNMTi were also reported for their inhibitory action against DNMT, including small molecules such as hydralazine and procainamide, natural molecules like psammaplins (bisulfide bromotyrosines) derived from a marine sponge, (−)‐epigallocatechin‐3‐gallate (EGCG), the primary polyphenol obtained from green tea, catechol‐containing polyphenols like catechin, epicatechin and bioflavonoids like quercetin, genistein and fisetin.^107^, ^108^, ^109^, ^110^, ^111^

During unusual conditions, continuous expression of DNMTs results in DNA hypermethylation which plays a vital role in cancer occurrence and building drug resistance.^112^, ^113^ Depletion of this enzyme using nucleoside deoxycytidine analog DAC as a DNMTi is the most widely used gene hypomethylating or demethylating agent; thereby, it is used as a monotherapeutic agent for cancer treatment.^114^, ^115^, ^116^, ^117^ However, the major limitation of DAC is its instability due to its short half‐life which resulted in a transient effect against depletion of DNMT1.^118^, ^119^, ^120^ To overcome this, Vijayaraghavalu et al. reported that DAC‐loaded nanogel formulation delivers DAC in the intracellular space efficiently and sustains the drug effect, leading to prolonged depletion of DNMT1.^18^ Authors formulated four different DAC nanogel formulations (NG‐70, NG‐80, NG‐85, and NG‐100), which showed a better and prolonged antiproliferative effect than the DAC solution alone in both MCF‐7/Adr cells (doxorubicin‐resistant) and MCF‐7 cells (doxorubicin‐sensitive). Furthermore, DAC nanogel formulations exhibit a more sustained drug release and significant antiproliferative effects in both decitabine DAC‐resistant and sensitive B16 melanoma cells compared to DAC in solution. Moreover, the DAC nanogel (NG‐70) exhibited an astounding cytotoxicity effect with an IC_50_ of 24‐fold and 8‐fold lesser concentration than the DAC solution upon treating B16‐resistant and sensitive cells. A similar result was observed in the cell cycle analysis.^18^ Another study by Briot et al. emphasizes the outcome of an improved pharmacokinetic property, oral bioavailability, and therapeutic index of DAC delivered by DAC‐loaded lipid nanocapsules (LNCs) for acute myeloid leukemia (AML).^121^ In vitro release of DAC‐loaded LNCs formulated with co‐mixture Transcutol® HP and Tween® 80 (THP‐T80‐LNCs) showed a sustained drug release (70%) within 24 h. Besides, DAC‐loaded THP‐T80‐LNCs showed improved cytotoxic effect with lesser IC_50_ (670 nM) on non‐resistant human erythroleukemia (HEL) cells as compared to the free drug (1000 nM) and blank THP‐T80‐LNCs.^121^ A study by Neupane et al. showed remarkable results on the enhanced bioavailability of lipid drug‐conjugated (LDC) DAC compared to DAC suspension through oral administration.^122^ In vitro release study revealed that LDC‐DAC displayed burst release (26.34% ± 1.36%) at the initial 2 h followed by sustained release (76.69% ± 2.657%) till 24 h. This biphasic release pattern of LDC‐DAC nanoparticles is precisely contrary to the suspension of DAC, which showed 96.45%–99.78% of drug release at the initial 4–8 h. Additionally, LDC‐DAC nanoparticles exhibited improved permeation when compared with the DAC solution.^122^

Likewise, the delivery strategy of AZA through unilamellar vesicular liposome (lyophilized) prepared by lipid thin film hydration method was reported by Kesharwani et al., to overcome the clinical limitations of the drug.^15^ AZA is a highly water‐soluble hydrophilic drug, with fast metabolism, poor bioavailability, and short half‐life (<4 h).^123^ In addition, it shows low permeability at the specific targeted cancer site and nonspecific distribution. Liposomal drug delivery is one of the most promising approaches and can incorporate AZA, shows a prolonged circulation time and higher drug encapsulation capacity with the maximum delivery ability to the target site.^124^, ^125^, ^126^, ^127^ In vitro release studies at different pH showed a sustained release (74 ± 2.3%) of the drug up to 36 h, as a liposomal formulation of AZA whereas free AZA showed 90% release in 2 h. Further, the hemolytic toxicity study showed that AZA liposome was less toxic for red blood cells (RBC) than free AZA. Cytotoxicity assay on MCF‐7 demonstrates more cytotoxicity when treated with AZA liposome than free AZA.^15^ In another study, a novel prodrug of EGCG (pEGCG) have been synthesized and studied on human breast cancer MCF‐7 and MDA‐MB‐231 cells where pEGCG showed dose and time‐dependent proliferation which was not obtained in MCF10A cells (control). Furthermore, both EGCG and pro‐EGCG repressed the catalytic subunit of telomerase which is transcription of human telomerase reverse transcriptase (hTERT), via epigenetic mechanisms in estrogen receptor (ER)‐negative MDA‐MB‐231 cells and ER‐positive MCF‐7 cells. The downregulation of hTERT expression was observed due to hypomethylation and histone deacetylations of hTERT promoter, facilitated at least partially by inhibiting the function of DNMT and HAT respectively.^128^ Different delivery strategies for DNMTi are shown in Table 1.

**TABLE 1:** Delivery strategies of DNA methyltransferase inhibitors (DNMTi).

Epigenetic modulators (DNMTi)	Delivery systems/strategies	Particle size (PS) and zeta potential (ZP)	Disease	Cell lines	Outcomes	References
Decitabine (DAC)	Nanogel (NG‐70, NG‐80, NG‐85, and NG‐100)	PS: DAC‐NG‐70: 244 nm ZP: DAC‐NG‐70: −19 ± 1.0	Cancer	MCF‐7/Adr, MCF‐7, DAC sensitive, and resistance B‐16 cells	1. NG‐70 exhibited a more prolonged cytotoxic effect than other nanogel. 2. Improved antiproliferative effects with lesser IC_50_ with the DAC‐NG‐70 than solution of DAC.	[18]
DAC	Lipid nanocapsules (LNCs)	PS: 26.0 ± 0.4 nm	Acute myeloid leukemia (AML)	Nonresistant HEL AML cells	1. Improved cytotoxic effect with lesser IC_50_ (670 nM) of DAC‐LNCs as compared to free drug (1000 nM).	[121]
DAC	Lipid drug‐conjugate (LDC)	PS: 202.6 ± 1.65 nm ZP: −33.6 ± 0.845 mV	–	–	1. LDC‐DAC showed burst release (26.34% ± 1.36%) at the initial 2 h followed by sustained release (76.69% ± 2.657%) till 24 h. whereas suspension of DAC showed 96.45%–99.78% of drug release at the initial 4–8 h.	[122]
Azacytidine	Unilamellar vesicular liposome	PS: 107 ± 1.1 nm ZP: −25 mV	Cancer	MCF‐7	1. AZA‐liposome showed sustained release (74 ± 2.3%) of the drug up to 36 h as compared to free AZA (90% release in 2 h). 2. AZA liposome showed more cytotoxic effect (23.23 μM) than free AZA (39.54 μM).	[15]
Prodrug of epigallocatechin‐3‐gallate (pEGCG)	Prodrug	–	Breast cancer	ER‐negative MDA‐MB‐231 and ER‐positive MCF‐7 cells	1. p‐EGCG inhibited and downregulated the transcription of human telomerase reverse transcriptase (hTERT).	[128]
Genistein	Lactalbumin nano‐formulations (LANPs)	PS: 100–150 nm ZP: −25 to −30 mV	Oral squamous cell carcinoma	JHU011 cells	1. More decrease in cells viability by GLANPs (~60%) than free genistein (~40%). 2. Approximately 3.5‐fold ROS generation increased by GLANPs in cells compared to free GEN. 3. Increased ubiquitin expression which activates proteasomal degradation resulted in EZH2 degradation.	[129]

### 4.2 Histone modifiers

Histone modifiers are classified based on the inhibition processes of different enzymes associated with the epigenetic mechanism. Commonly known histone modifiers are histone acetyltransferase inhibitors (HATi), histone deacetylase inhibitors (HDACi), and histone methyl transferase inhibitors (HMTi). Among these, histone deacetylase inhibitors (HDACi) are extensively studied and their delivery strategies are well explored.

#### 4.2.1 Histone acetyltransferases inhibitors

Histone acetyltransferase inhibitors (HATi) are the potential compounds that inhibit the catalytic activity of HATs; thus, becoming a promising approach in the treatment of different cancers and other diseases including cardiac hypertrophy, Alzheimer's (AD), diabetes, asthma, and hyperlipidemia.^130^, ^131^, ^132^, ^133^ However, to date, very few HATi have been reported. Bi‐substrate analog (e.g., Lys‐CoA and H3‐CoA‐20) that first came across as a HATi inhibits HATs p300 and PCAF selectively and mediates the re‐expression process of tumor suppressor genes in cancer.^134^, ^135^, ^136^ In addition, various naturally occurring compounds have been reported as HATi, such as anacardic acid from cashew nut shell liquid, curcumin (diferuloylmethane) from *Curcuma longa* rhizome and garcinol, a polyisoprenylated benzophenone derivative from *Garcinia indica* fruit rind. Among these natural products, curcumin inhibits H3 and H4 acetylation by HATs P300 and CPB, whereas anacardic acid and garcinol show inhibitory activity by HATs p300 and PCAF with low potency.^137^, ^138^, ^139^ Recently, small molecules have also been reported as HATi, including a group of isothiazolinones, a series of quinoline derivatives, and garcinol analogs due to their inhibitory property on either or both HATs p300 and PCAF.^140^, ^141^, ^142^, ^143^ The delivery strategy of HATi has not been extensively exploited yet with a perspective of modulating the epigenome, which also needs attention in the future. Although different cargos, including liposomes, polymeric nanoparticles, conjugates, micelles, and dendrimers for curcumin, are reported in the literature, these have still not yet been studied in epigenetic modulation to inhibit HATs.

#### 4.2.2 Histone deacetylase inhibitors

Histone deacetylase inhibitors (HDACi), widely diverse epigenome modulators, play a significant role in epigenome‐regulated gene expression. Briefly, HDACi inhibits the deacetylation process of histone and induces acetylation or hyperacetylation, which increases the gene transcription. HDACi can be classified into different groups depending on their structure, such as short‐chain fatty acids, hydroxamic acids, cyclic tetrapeptides (with and without epoxyketone group), and benzamides.^144^ Short‐chain fatty acids such as valproic acid (VPA), butyrate, 4‐phenylbutyrate, and sodium phenylbutyrate exhibit their mode of action based on the carboxylic group, which can occupy the acetate escaping tunnel and are also involved in zinc‐binding function or compete with the acetate group released in the deacetylation reaction.^145^

These small molecules, either solely or in combination with other drugs, are subjected to several clinical trial phases (phases 1 and 2) for the treatment of different types of cancers, including cervical and breast cancers, AML, leukemia, non‐small cell lung cancer (NSCLC) and solid tumors, HIV infection, Huntington's diseases, and amyotrophic lateral sclerosis.^146^, ^147^, ^148^, ^149^, ^150^, ^151^, ^152^ Although VPA is a well‐known anticonvulsant agent, clinical trials (Phase 1 and 2) have begun as HDACi drugs for treating lymphocytic leukemia.^153^, ^154^ In addition, several reports stated combinational therapies such as VPA and AZA for the treatment of AML and myelodysplastic syndromes while combination of hydralazine and magnesium valproate for cervical and breast cancers, and so forth.^146^, ^155^, ^156^

Likewise, Novohradsky et al. reported platinum valproic acid conjugate [Pt (IV)‐VPA] wherein one or two VPA as axial ligands were conjugated with the cisplatin derivative Pt (IV), [cisPt (Ac)(VPA)], and [cisPt (VPA)_2_] for the treatment of human ovarian cancer.^157^ More precisely, the platinum (Pt) complex is well known to kill cancer cells as it binds with the DNA and denatures it followed by apoptosis or necrosis.^158^ Besides, HDACi are involved in the hyperacetylation process of histones that may promote ease of access of DNA within chromatin structure as the interaction between DNA and histone protein becomes weakened; hence, DNA damaging activity is enhanced by platinum along with other drugs involved in DNA damaging process while HDACi is administered simultaneously.^159^, ^160^, ^161^ Therefore, the authors developed a conjugate containing prodrug of Pt (IV) and VPA that allows the release of an active component of Pt (II) at effective concentration after reaching the destination site.^157^ In addition, hydroxamic acids exhibit a promising approach as HDACi for treating different human diseases, including cancers. Hydroxamic acids such as Vorinostat (suberoylanilide hydroxamic acid, SAHA/VOR), Panobinostat (LBH589), CHR‐3996, CHR‐2845, SB939, ITF2357 (Givinostat), PXD101 (Belinostat), and JHJ‐26481585 proved their inhibitory property against HDAC enzymes.^162^, ^163^, ^164^, ^165^, ^166^, ^167^, ^168^ Further, MS‐275 (SNDX‐275, Entinostat) and MGCD0103 (Mocetinostat), known as benzamides, are in phase 1 and 2 of clinical trials for the treatment of hematological diseases such as leukemia along with solid neoplasms.^169^, ^170^

**FIGURE 5:**
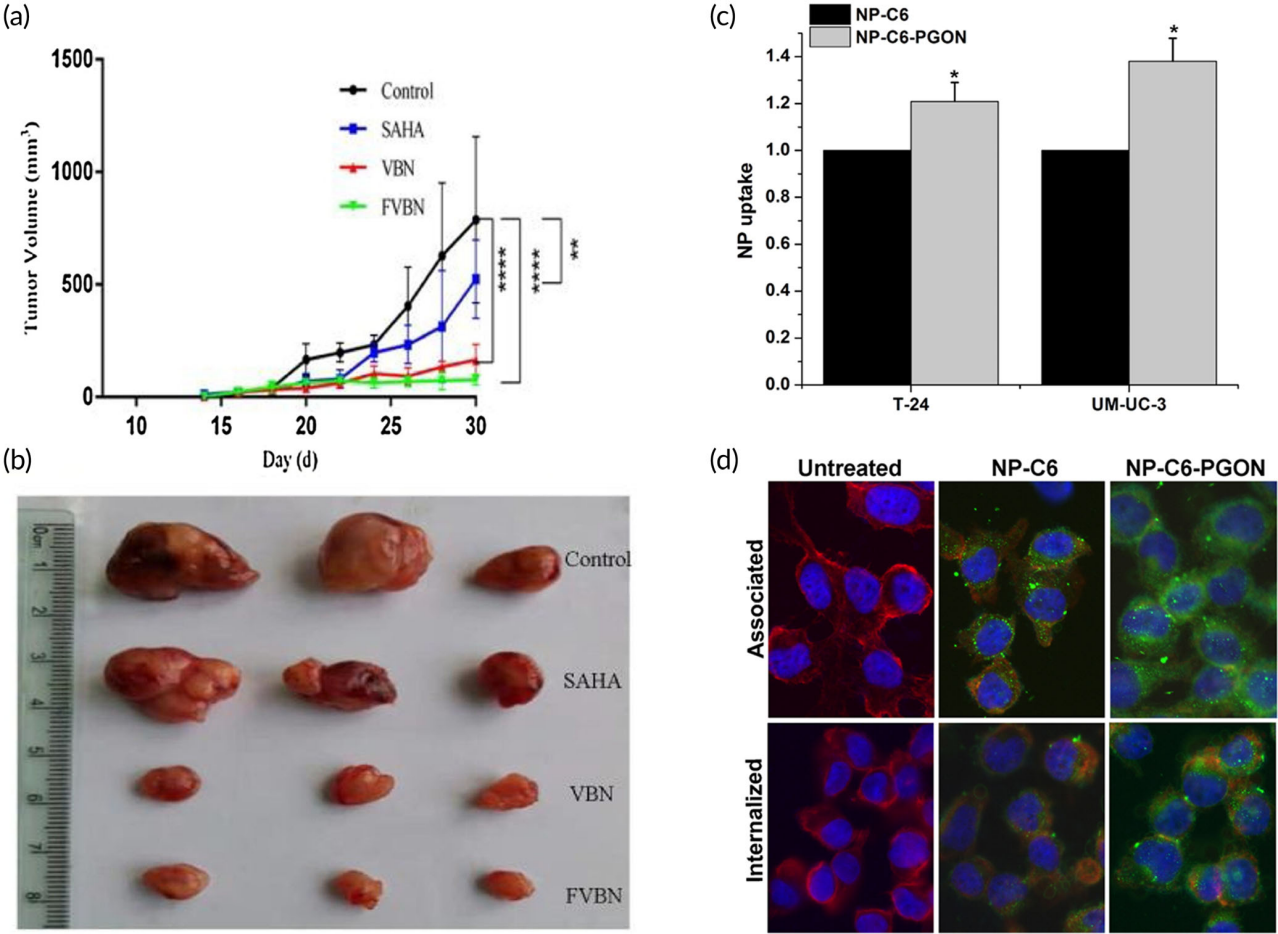
In vivo anti‐tumor efficacy by folate bound bovine serum albumin nanogel incorporating vorinostat (SAHA) (FVBNs) and in vitro cellular uptake by belinostat loaded poly(guanidinium oxanorbornene) modified PLGA nanoparticles (NP‐Bel‐PGON). (a) Tumor volume (mm^3^) curve compared with treatment upon control group, Free SAHA group, blank nanogel (VBN), and FVBNs. (b) Pictorial representation of ovarian tumor compared with treatment upon control group, SAHA, VBN, and FVBN. (c) FACS analysis indicated enhanced cellular uptake by NP‐C6‐PGON compared with NP‐C6 treatment upon bladder cancer cells T‐24 and UM‐UC‐3. (d) Fluorescence microscopy result showed cellular uptake of NP‐C6 and NP‐C6‐PGON incorporating C6 (green) in the cytoplasm of T‐24 cells. The nucleus was shown in blue (stained with DAPI) and cell membrane was shown in red (stained with Texas Red X‐phalloidin). Figure 5a,b reprinted with permission from Guo et al.^176^ and Figure 5c,d reprinted with permission from Martin et al.^179^

Vorinostat (VOR/SAHA; brand name: Zolinza™, Merck) was the first approved drug as HDACi for cutaneous T‐cell lymphoma by US FDA. Further, in vivo study in leukemic rodent model using VOR has shown a satisfactory effect by means of enhanced survival rate along with remarkable in vitro data.^171^, ^172^, ^173^, ^174^ In addition, VOR is a hydrophobic drug containing a neutral charge and showing approximately 71% protein binding efficiency; hence, these all may lead to promote noncovalent binding with the hydrophobic sites of a lipophilic amino acid chain of a polypeptide resulting in improving release and cellular uptake. Besides, the albumin nanoparticle has been proven as a clinically successful delivery system that possesses several advantages, such as greater cellular uptake by tumor cells, lack of toxicity, and ease of availability as compared to other nanocarriers.^175^ Therefore, Chandran et al. developed albumin‐based nanomedicine system for the intravenous delivery of VOR for the treatment of AML. This VOR encapsulated albumin nanoparticles (~100 nm) showed increased cellular uptake with significantly lower IC50 in AML cell lines as well as in patient samples, and encouraged enhanced HDAC inhibition, cell cycle arrest oxidative injury, and apoptosis as compared to free drug.^91^ In addition, Guo et al. reported surface functionalized delivery strategy where folate‐bound bovine Serum albumin nanogel incorporating VOR (FVBNs) for tumor targeting chemotherapy showed improved solubility, stability, cellular uptake, and promoted to lipase‐responsive release of the drug.^176^ In vitro study on cells including HUVEC, B16‐F10, and A2780 showed enhanced cytotoxicity with decrease IC_50_ from 29.33 to 10.64 μM, from 0.32 to 2.14 μM and from 17.23 to 9.43 μM, respectively, by FVBNs treatment as compared to free VOR. Further, in vivo experiment by FVBNs against nude mice bearing solid ovarian cancer, revealed excellent antitumor effect without liver damage (Figure 5a,b).^176^ Another research group reported pH‐responsive diaryltriazolyl‐based prodrugs for HDACi for the treatment of cancer to avoid existing limitations of HDACi, such as rapid metabolism as well as clearance and toxicity. Both the HDACi, such as CI‐994 and SAHA have been chosen for this study due to their clinical importance. Dose‐dependent and kinetics restoration of HDAC inhibition were determined by bioluminescence resonance energy transfer (BRET) assay, which revealed satisfactory data.^177^ Apart from this, ample delivery strategies for VOR such as polymeric nanoparticles, polymeric micelles, hydrogel system, lipid‐based nanoparticles, inorganic nanoparticles, prodrugs, and conjugates are also reported by various research groups which have also been reviewed recently by Le et al. and summarized in Table 2.^178^

**TABLE 2:** Delivery strategies of histone modifiers.

Epigenetic modulators	Delivery systems/strategies	Particle size (PS) and zeta potential (ZP)	Disease	In vitro (cell lines) and/or in vivo models	Outcomes	References
VOR	Hyaluronic acid decorated solid lipid nanoparticles (SLN)	PS: 102.3 ± 1.6 nm ZP: −9.0 ± 0.7 mV	Cancer	SCC‐7 and MCF‐7 cells	1. More cytotoxic than the native form of drug and VOR‐SLNs. 2. Prolonged blood circulation, decreased VOR clearance rate in rats, conferring higher plasma concentration, and bioavailability.	[195]
VOR	SLN	PS: 86.5 ± 4.5 nm ZP: −22.2 ± 0.5 mV	Cancer	MCF‐7, A‐549 and MDA‐MB‐231	1. Bi‐phasic release pattern, in initial 2 h burst release (25%) followed by sustained release (35%) till 24 h. 2. Improved plasma circulation time, AUC, and decreased elimination rate constant.	[196]
VOR	Nanostructured lipid carriers (NLCs)	PS: ~150 nm ZP: ~22 mV	Cancer	MCF‐7, A‐549, and SCC‐7 cells	1. Sustained in vitro release profile, and enhanced cellular uptake with improved cytotoxic effect. 2. AUC was increased ~4.4‐fold by VOR‐NLCs as compared to free VOR.	[197]
VOR	Poly(dl‐lactide‐co‐glycolide) nanofiber coated stent	–	Cholangiocarcinoma	HuCC‐T1, SNU478, SNU245, SNU 1196 CCA cell lines and HuCC‐T1‐bearing nude mice	1. Induced acetylation of histone H4 and inhibited in vitro and in vivo expression of histone deacetylases 1, 3, 4, 5, and 7. 2. Higher inhibition rate of tumor growth than VOR injections in HuCC‐T1 bearing mice.	[198]
VOR	Poly(dl‐lactide‐co‐glycolide)/poly(ethylene glycol) copolymer based nanoparticles	PS: <100 nm	Cholangiocarcinoma	HuCC‐T1 cells and HuCC‐T1 bearing xenograft model in nude mice	1. Increased acetylation of histone‐H3 with anti‐tumor efficacy in vivo. 2. Repression of the expression of histone deacetylase, mutant type p53, p21, and PARP/cleaved caspase‐3.	[90]
VOR	PEG‐PLGA‐micelles	PS: 124.06 ± 2.6 nm	Solid tumors	B16F10, MDA MB 231 cells, and B16F10 tumor‐bearing mice	1. Superior cellular internalization, enhanced cytotoxic activity, and greater apoptosis with 54.9% cells killing efficiency compared to free drug (36%) after 24 h. 2. Compared to free VOR‐treated animals, 1.78‐fold reduction in tumor volume.	[199]
VOR	Poly(ethylene glycol)‐block‐poly(caprolactone) (PEG‐PCL) micelles	PS: ∼130 nm	Cancer	B16F10, MDA MB‐231 cell line and female C57BL/6 mice‐bearing tumor	1. Efficient cell cycle arrest in G2/M phase resulting in higher rate of apoptosis compared to free VOR. 2. Significant tumor suppression compared to free SAHA.	[94]
VOR	Poly(ethylene glycol)‐b‐poly(dl‐lactic acid) (PEG‐b‐PLA) micelles	PS:75.67 ± 7.57 nm and 87.33 ± 8.62 nm for drug to nanocarrier ratio 1:10 and 1:15 respectively	Cancer	–	1. Significant increase in mean residence time, serum half‐life, and area under curve. 2. Reduced VOR clearance, particularly after intravenous dosing.	[200]
VOR	Pluronic F127 (PF127)‐VOR micelles	PS: 91.88 ± 10.70 nm	Cancer	HepG2, Caco‐2, MCF‐7 cell lines, and Ehrlich ascites carcinoma cells (EAC) bearing female Swiss albino mice	1. More cytotoxic effect as compared to free drug. 2. Inhibiting tumor growth as well as exhibiting significantly reduced hepatic and renal toxicity after intravenous administration.	[201]
VOR	Mesoporous silica nanoparticles (MSNs)	PS: ~140–200 nm ZP: +20 mV	Cancer	HCT116 and cutaneous T‐cell lymphoma cell line (HH)	1. Permeability was enhanced 4‐fold as compared to free drug. 2. Significantly induced histone hyperacetylation and expression of HDACi‐target genes, and induced extensive apoptosis.	[202]
VOR	D‐α‐tocopheryl polyethylene glycol 1000 succinate‐coated liposomes (TPGS‐VOR‐LIPO)	PS: 176.99 ± 2.06 nm ZP: −26.3 ± 0.8 mV	Breast cancer	MCF‐7 cells	1. Sustained release of VOR over 48 h. 2. Improved cellular uptake qualitatively and quantitively with superior cytotoxic effect as compared to noncoated liposome and free VOR solution.	[203]
SAHA	Redox responsive nanoformulation (SAHA‐S‐S‐VE/TPGS NF)	PS: 148 nm	Solid tumor	MDA‐MB‐231, PC‐3, HepG2, A549 cells, and H22‐bearing Kunming mice	1. Significant improvement in in vitro anticancer activity. 2. Accumulated in the tumor area and efficiently inhibited the tumor growth.	[204]
VOR	Folate‐bound bovine Serum albumin nanogel	PS: ∼20 nm	Tumor	A2780, B16–F10, HUVEC cells, and B16–F10 cells bearing mice melanoma models	1. Growth of tumor cell was blocked in G1/G0 phase. 2. Excellent antitumor effect without liver damage.	[176]
VOR	Mixed‐matrix hydrogel (HPMC/HPC)	–	Cutaneous T‐cell lymphoma (CTCL)	–	1. Higher AUC than oral application with higher relative bioavailability. 2. It achieved sustained permeation of VOR through skin for 24 h in vivo along with no damage, swelling, and inflammation after topical administration.	[205]
VOR	pH‐responsive polymer conjugate	Tumor	Mesothelioma tumors	MeT‐5A, Meso13, Meso34, Meso56 cells, and AK7 murine mesothelioma cells bearing mice	1. Significantly decreased cancer cell viability. 2. Histone H3 acetylation was increased in tumors of treated mice	[206]
Octanoato (OA)	Pt(IV) based prodrug: Pt(IV)diOA	–	Tumor	A2780, A2780cisR, MCF‐7, HCT‐116 cells, and Syngeneic murine model of lung cancer using Lewis lung carcinoma cells	1. Platinum content in DNA was more as compared to other treatments. 2. Enhanced cellular uptake, and downregulation of HDAC with improved antitumor activity in in vivo.	[207]

Other than Vorinostat, a few delivery strategies of Belinostat and Panobinostat have also been explored by different research groups. For instance, Martin et al. fabricated belinostat‐loaded PLGA nanoparticles (NP‐Bel‐PGON) which was surface modified with a novel cell penetrating polymer, poly(guanidinium oxanorbornene) (PGON; Figure 5c,d). In in vitro study, treatment with NP‐Bel‐PGON on bladder cancer cell lines (T‐24, UM‐UC‐3, and RT‐4) showed significant decrease in IC_50_ with sustained hyperacetylation as compared to free belinostat. Further, engrafted tumor mice model demonstrated a 70% reduction in tumor volume after intratumorally injected with NP‐Bel‐PGON along with 2.5‐fold increase in intratumoral acetylation in histone H4, when compared to blank NP‐PGON.^179^ In another report, Belinostat loaded lipid polymer‐based hybrid nanoparticulate (LPN) system for the treatment of breast cancer has been reported. In vivo study demonstrated multi‐fold increase in the pharmacokinetic parameters by drug‐loaded LPN in tumor bearing animals as compared to the free Belinostat along with 20‐fold enhanced uptake and retention of the drug‐loaded LPN in tumor tissues of the animal model at the end of 4 h.^180^ Further, very poorly water‐soluble HDACi, Panobinostat (PAN) loaded Poloxamer 407 (P407) based nano‐micelles have been demonstrated for the treatment of high‐grade glioma. Convection‐enhanced delivery (CED) of PAN‐loaded nano‐micelles showed in vitro cytotoxic effect on human (U87‐MG and M059K) and rat (F98) glioma cells in a dose‐dependent manner and improved survival rate of animals compared with control group after in vivo CED administration.^93^

In 2009, a cyclic tetrapeptide without epoxyketone group named Romidepsin (Depsipeptide or FK228) obtained from *Chromobacterium violaceum*, was approved by the US FDA as HDACi for the treatment of cutaneous T‐cell lymphoma. The mechanism of Romidepsin involves in releasing thiol in the cells due to its interaction with the disulfide bond. Further, this thiol's interaction with the zinc atom at the enzyme binding site interferes with the activity of zinc‐dependent HDAC enzymes resulting in inhibiting the activity of zinc‐dependent HDAC enzymes.^181^ O'Connor et al. synthesized nanoromidepsin (NR) particles by using specific drug/polymer/surfactant ratio. NR particles exhibited improved cytotoxicity and apoptosis along with increased acetylation in histone H3/H4 in a concentration and time‐dependent manner when compared with native Romidepsin. Further, in vivo pharmacokinetic study demonstrated that intraperitoneal and intravenous administration of NR showed 3‐fold and 25‐fold increase in AUC, respectively, as compared to Romidepsin.^182^

Additionally, Sabbatini et al. reported a bifunctional prodrug Pt (IV) containing HDACi as an axial ligand named 2‐(2‐propynyl) octanoato for the treatment of colon cancer.^20^ Here, authors synthesized cyclohexane‐1*R*,2*R*‐diamine (dach) based compound ([Pt (dach)]^2+^) like (OC‐6‐44)‐acetatodichlorido(cyclohexane‐1*R*,2*R*‐diamine)(rac‐2‐(2‐propynyl)octanoato)platinum(IV), namely 1, and its isomer such as (OC‐6‐44)‐acetatodichlorido‐(cyclohexane‐1*R*,2*R*‐diamine)(2*R*‐(2‐propynyl)octanoato)‐platinum(IV), namely 1R, and (OC‐6‐44)‐acetatodichlorido‐(cyclohexane‐1*R*,2*R*‐diamine)(2*S*‐(2‐propynyl)octanoato)‐platinum (IV), namely 1*S* and also compared among them along with reference compounds rac‐POA, cisplatin, oxaliplatin, [PtCl_2_(dach)], and the racemic cisplatin‐based derivative (OC‐6‐44)‐acetatodiamminedichlorido(2‐(2‐propynyl)octanoato)platinum(IV), namely 2. In vitro study in human colon cancer cell lines (HCT 116, SW480, and HT‐29) and mouse colon cancer cell lines (CT26) revealed that IC_50_ of both the isomers 1*R* and 1*S* was similar to the compound 1. Further, in vivo study was performed in BALB/c mice having CT26 colon cancer by administering compound 1 along with oxiplatin by oral as well as intravenous route. Intravenous administration of compound 1 showed a more efficient as well as promising approach than oral delivery in terms of higher antitumor effect alongside lesser toxicity and side effects.^20^ In another report, Chiu et al. stated that curcumin liposomal formulation named as Lipocurc™ which can also inhibit HDAC, showed anti‐apoptotic and enhanced motor deficits or neurotropic effects when it was administered intravenously in DJ‐1 gene knockout (KO) at Park‐7 gene locus of rat model of Parkinson's disease (PD).^183^

#### 4.2.3 Histone methyl transferase inhibitors

Histone methyl transferase inhibitors (HMTi) are less explored as compared to HDACi and DNMTi. S‐adenosylmethionine (SAM) and different analogs of SAM, including S‐adenosylhomocysteine (SAH), 5′‐methylthioadenosine (MTA), and sinefungin are well known as HMTi. These inhibitors also act as a cofactor for other enzymes such as DNMT.^184^ In addition, a fungal mycotoxin named chaetocin has been reported for its inhibitory effect on G9a; however, it fails to inhibit other KMTs, including SET7/9 or E(Z). Further, selective inhibitors of G9a, such as acyl benzimidazole, for example, BIX‐01338 and BIX‐01294, were reported without inhibitory activity against Suv39H1 or PRMT1. In vitro study exhibited that treatment with BIX‐01294 led to decreased demethylation of H3K9, whereas the mono and trimethylation remained unaffected along with other methylation sites, H3K27 or H4K20.^185^, ^186^

Additionally, UNC0224, a potent and relatively selective inhibitor for the G9a, was reported by Liu et al. shows also selectivity against SET7/9 (a H3K4 HMTase) and SET8 (a H4K20 HMTase).^187^ Recently, a cyclopentenyl analog, 3‐Deazaneplanocin A (DZnep), was reported for its inhibitory ability against *S*‐adenosyl‐l‐homocysteine hydrolase.^188^, ^189^ Further, it also inhibits trimethylation of lysine 27 on H3 (H3K27me3) and lysine 20 on H4 (H4K20me3), which was proved when treated on human acute myeloid leukemia cells (HL‐60 and OCI‐AML3 cells) and involves in depletion of EZH2.^190^ AMI‐1 and its derivatives, AMI‐2 to AMI‐6 are known as inhibitors of PRMT with minimal inhibitory effect on KMTs. Moreover, these inhibitors are in the preclinical testing stage as they show low specificity towards HMT enzymes and create toxicity in different cell lines. On the other hand, in an in vivo study, tranylcypromine (2‐PCPA) which showed inhibition against histone demethylating enzymes, LSD1 showed IC_50_ value of 20.7 mM, is not considered as selective for LSD1 due to its inhibitory activity against both MAO‐A and MAO‐B enzymes irreversibly (IC_50_ 2.3 and 0.95 mM, respectively). Thus, tranylcypromine has not yet been involved in clinical trials because of its collateral effects.^191^

The delivery strategy of HMTi is still at its infant level. Recently, Zeybel et al. reported the delivery strategy of DZnep through a surface‐modified liposome where the liposome surface was bearing antibodies for the active target of liver myofibroblasts.^17^ More precisely, authors proved that the epigenetic modulator, DZnep loaded immunoliposome (antibody‐liposome), could repress the H3K27me3 epigenetic mark of hepatic myofibroblasts selectively while inhibiting EZH2 and other HKMTS enzymes resulting in suppressing the progression of fibrosis in liver and reduces the risk of liver damage. In vivo study exhibited that DZNep‐loaded C1–3 ScAb liposomes promote to loss of the H3K27me3 epigenetic mark present in myofibroblasts in the liver due to the targeting property, whereas the H3K27me3 epigenetic mark was still there when CSBD9/DZNep liposomes (control) were treated by intravenous (i.v.) injection.^17^

#### 4.2.4 Histone ubiquitination

A few research groups have reported their investigations on the delivery strategies of epigenetic modulators that function through histone ubiquitination‐based pathways. Recently, a study organized by Sardoiwala et al. stated that chitosan nanocarriers encapsulated with fingolimod (FTY720) hydrochloride as an efficient neuroprotective protein phosphatase 2 activator (PP2A). The FTY720 loaded chitosan nanoformulations have been tested on in vitro and ex vivo Parkinson's disease (PD) models and exhibited improved neuroprotective effect and bioavailability as the chitosan is much biocompatible. The research findings proved that FTY720‐loaded chitosan nanocarriers have functioned on ubiquitination and depletion of phospho‐serine 129 α‐Syn mediated by the interaction of PP2A and PcG protein‐EZH2 for regulating epigenetic mechanisms involved in PD prevention.^92^

Similarly, another research by Sardoiwala et al. demonstrated the use of metformin‐loaded bio‐compatible polydopamine nanoformulation for treating PD by upregulating EZH2 mediated ubiquitination and proteasomal degradation of aggregated pSer129 α‐Syn pathway.^192^ A study by Kushwala et al. illustrates that encapsulation of PRT4165 in human serum albumin nanoparticles (HASNps) showed better therapeutic efficacy by proving enhanced apoptosis, depolarized mitochondrial membrane, sub‐G1 cell cycle arrest, reactive oxygen species (ROS) generation, and caspase 3 activations. The nanoformulation represses the Bmi1 via the ubiquitin‐proteasome pathway (UPP) and regulated H2AK119 ubiquitination in AML cells. Further, in the AML xenografted nude mice model, PRT4165 encapsulated HSANPs showed enhanced in vivo biodistribution, enhanced dispersibility, and biocompatibility, in turn positively influencing the suppression of leukemia stem cell marker, CD45^+^, and activation of myeloid monocytes differentiation marker, CD11b^+^.^193^ Similar group performed research on HASNPs encapsulated with an EZH2‐specific inhibitor, EPZ011989, that facilitates an efficient drug loading and sustainable release, thus increasing cellular uptake and nuclear localization of nanocomposite in human AML cell lines. In addition, this nanocomposite showed effective cytotoxicity and inhibitory effect on colony formation, and suppression of polycomb group (PcG) proteins such as EZH2, Bmi1, and so forth. This downregulation is associated with reduced H3K27me3 and H2AK119ub modifications indicating chromatin compaction. Further studies revealed EZH2 and c‐Myb downregulation via ubiquitination. Similarly, in engrafted nude mice, reduced expression of CD11b^+^ and CD45^+^ was observed. These experimental findings provide valuable insights into targeted epigenetic therapy of AML.^194^ Various reported delivery strategies of histone modifiers are shown in Table 2.

## 5 BIOMACROMOLECULES

Biomacromolecules including oligonucleotides, siRNA, miRNA, and CRISPR/Cas system have shown promising attributes as an emerging therapeutic tool for regulating the epigenetic status of a gene. Amato et al. reported that a 20‐base pair antisense oligonucleotide, MG98 is a potential human DNMT1 inhibitor that binds at the 3′ untranslated site of DNMT1, which in turn results in transcription inhibition.^208^ MG98 appeared suitable for treating metastatic renal carcinoma (MRC) when subjected to combinational therapies with a chemotherapeutic drug, Roferon‐A.^209^, ^210^ Trivedi et al. developed self‐assembled hyaluronic acid‐based poly(ethylene imine) (HA‐PEI) and HA‐poly(ethylene glycol) (HA‐PEG) blend nanoparticles for the delivery of miR34a RNA in A549 human lung adenocarcinoma epithelial cell lines. In both wild‐type A549 and cisplatin‐resistant A549 DDP cells, global nuclear DNA (nDNA) methylation status has been measured by enzyme‐linked immunosorbent assay‐like reaction (ELISA) using monoclonal antibodies such as anti‐5‐methylcytosine and anti‐5‐hydroxymethylcytosine. This study revealed that ~25% decrease in 5‐mC levels after 24 h of treatment of miR34a‐loaded HA‐NPs (50 nM) whereas no significant changes were observed post‐treatment of free miR34a and blank HA‐NPs. Likewise, methylation status of Global mitochondrial DNA (mtDNA) for both the cell lines was determined where ~30% 5‐mC level was found to be decreased 24 h of post treatment of miR34a encapsulated HA‐NPs (50 nM). However, 20% increase in 5‐hmC status was observed when compared to the untreated cells. Moreover, treatment group of free miR34a and blank HA‐NPs exhibited no significant changes after 24 h of treatment.

**FIGURE 6:**
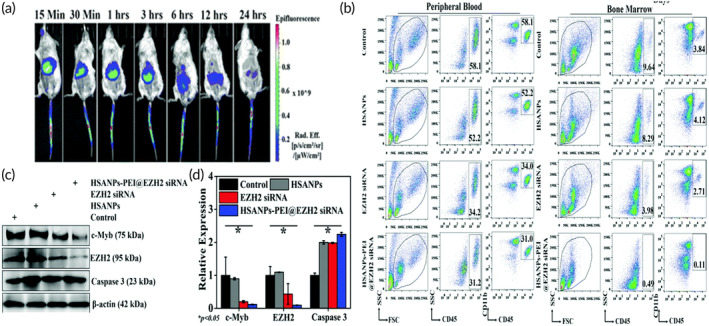
Results of in vivo biodistribution and in vivo efficacy. (a) Image of time‐dependent in vivo biodistribution analysis of Indocyanine green (ICG) tagged EZH2siRNA loaded human serum albumin nanoparticles (HSANPs‐PEI@EZH2siRNA). (b) In vivo efficacy analysis by HSANPs‐PEI@EZH2siRNA in acute myeloid leukemia xenografted model, wherein CD45 and CD11b markers from peripheral blood‐derived monocytes and bone marrow‐derived monocytes were analyzed using flow cytometry. (c) Western blot result for protein expression on spleen tissue and (d) Quantification of relative expression of different proteins (c‐Myb EZH2, Caspase 3 level, and Beta‐Actin). Reprinted with permission from Kaundal et al.^211^

Kaundal et al. reported human serum albumin (HSA) nanoparticle incorporating small interfering RNA (siRNA) for targeting EZH2‐expressing genes in AML.^211^ As HSA nanoparticles (HSANPs) are taken up more rapidly in the tumor site due to enhanced permeability and retention (EPR) effect and the tumor targeting properties of albumin receptors, resulting in increased amount of drug and gene reaching into specific site of tumor.^212^ Mostly, positive charge‐bearing HSANPs which is commonly prepared by conjugating polyethyleneimine (PEI), exhibited enhanced cell transfection efficiency during genetic material delivery.^213^, ^214^ Here, authors performed the cell viability study using developed cationized HSANPs loaded with EZH2siRNA (HSANPs‐PEI@EZH2siRNA) and free EZH2‐siRNA in both U937 and HL60 AML cells which revealed the IC50 values 29 ± 2.5 and 50 ± 10.11 nM in HL60 cells and 39 ± 15 nM and 60 ± 20 nM in U937 cells in 48 h, respectively. Later, protein and gene expression studies have shown downregulation of the EZH2 expression as well as lower levels of H3K27me3 Bmi1, H2AK119ub, and c‐Myb expression by HSANPs‐PEI@EZH2siRNA. Henceforth, it suggested the silencing of EZH2 which is governed by the regulatory function of c‐Myb which was also confirmed by the chromatin immunoprecipitation assay (ChIP). In vivo biodistribution exhibited accumulation of formulation in liver after 30 min without any offsite deposition (Figure 6a). Further, in vivo efficacy study in AML xenograft mice model demonstrated that treatment with HSANPs‐PEI@EZH2siRNA and free EZH2siRNA significantly reduced CD45+/CD11b+ in AML cells from 58.1% to 31% and 31.2%, respectively, in peripheral blood circulation and from 9.64% to 0.11% and 0.49% in bone marrow fractions, respectively (Figure 6b). Finally, AML cells in xenografted mice model showed downregulation of CD45+/CD11b+ expression which is also associated with expression of c‐Myb like in vitro study alongside downregulation of EZH2 and Caspase 3 level (Figure 6c,d).^211^

Recently, Acharya et al. reported that tannic acid‐crosslinked chitosan matrices as potential epigenetic modulators during tissue regeneration. Concisely, authors have developed chitosan scaffolds crosslinked with tannic acid and compared them with commonly used glutaraldehyde‐crosslinked scaffolds as a control for cytocompatibility study to assess the role of scaffolds on the cellular epigenetic status. After 5 days, enhanced F hypomethylation was observed in the MC3T3 fibroblast cells cultured on chitosan scaffolds crosslinked with tannic acid compared to glutaraldehyde‐crosslinked scaffolds. The tannic acid scaffold positively influenced the gene expression of osteocalcin and negatively impacted the cox‐2 compared with glutaraldehyde‐crosslinked scaffolds. These findings were the proof of concept that states that the functional properties of a scaffold are too involved in epigenetic marker regulation for the bone tissue regeneration process.^215^

CRISPR/Cas (clustered regularly interspaced short palindromic repeats) based epigenome editing emerges as an exciting and promising tool for epigenome editing as it focuses on site‐specific modifications in the target DNA, histones proteins, and the architecture of chromatin.^216^ Many researchers around the globe are exploring the full potential of the CRISPR/Cas tool. The emergence of genetically evolved nuclease‐null deactivated or dead Cas protein (dCas) shows outstanding epigenome editing efficiency. Its DNA binding ability, site‐specific at primer (1 nt) level, and chromatin rearrangement properties have made it an effective epi‐effector (EE).^217^, ^218^ EEs are epigenetic modifying proteins or enzymes conjugated with dCas9 protein to form a dCas9‐EE complex. This complex can be activated by coupling with a functional, single guide RNA (sgRNA). This entire functional conjugate can be examined and translated for epigenetic modification. Depending on the mechanism of action and nature, EEs are categorized as epigenetic modifiers (EM) and transcription modifiers (TM).^219^

EMs such as p300, TET1, LSD1, and DNMT3 are enzymes that promote the alteration of epigenetic markers. Whereas TMs are transcriptional factors that can act as either activator (Herpes simplex viral protein VP16, VP48, VP64, VP120) and transactivator domain of NF‐κB‐p65 (nuclear factor kappa B) or repressor (Krüppel associated box, KRAB), SID4x, WRPW, chromo shadow (CS), and so forth.^219^, ^220^, ^221^ Eventually, using these insights, a few research groups explored the epigenetic efficacy of dCas9‐EE complexes such as dCas9‐DNMT3A for DNA methylation, dCas9‐TET1 for DNA demethylation, dCas9‐p300, dCas9‐LSD1, and combination of three effectors likes dCas9‐VPR (VP64, p65, and Rta) and DNMT3ACD–DNMT3L–KRAB and so forth.^222^, ^223^, ^224^, ^225^, ^226^, ^227^, ^228^, ^229^, ^230^, ^231^

Albeit with plentiful research and progress on using the dCas9‐EE complex, no notable studies focused on better formulation and efficient delivery strategy. This crucial void should be addressed at a compelling pace/momentum. In conclusion, we hope this context will address the current research gaps and lime lights the need for a delivery strategy for epigenome editing‐based therapeutic arena.

## 6 DELIVERY SYSTEMS OF COMBINATION THERAPY OF EPIGENETIC MODULATORS

Combination therapy such as different types of epigenetic modulators, a combination of an epigenetic modulator along with other drugs and/ or chemotherapy, immunotherapy, photothermal therapy, and photodynamic therapy have been reported to achieve the goals in terms of controlling the disease condition by improving the efficacy of drugs with minimum side effects. Delivery strategies of combination therapy of different epigenetic modulators and other modalities are summarized in the following section based on the epigenetic‐dependent mechanisms and nature of epigenetic modulators.

### 6.1 Combination of DNMTi and HDACi

Anomalous situations of epigenetic status such as DNA hypermethylation and histone deacetylation simultaneously contribute to initiate and progress of several diseases including cancers. Additionally, HDACi and DNMTi have shown remarkable efficacy in reducing tumor burden and extending survival rate in mouse models, with no significant toxicity. Clinical trials involving patients with solid cancers and advanced hematological malignancies have reported well‐tolerated combinations of HDACi and DNMTi, suggesting promising activity in conditions such as refractory advanced non‐small cell lung cancer, myelodysplastic syndromes (MDS), and acute myeloid leukemia (AML). These findings collectively imply that targeting the aberrant tumor‐specific epigenetic program through combined treatment with DNMTi and HDACi holds therapeutic potential in multiple myeloma.^232^, ^233^, ^234^, ^235^, ^236^To control the disease condition, treatment of a combination of DNMTi and HDACi through an appropriate delivery strategy have been explored by research groups getting synergistic therapeutic effect of the drugs.^237^ Likewise, Kim et al. investigated that epigenetic drugs such as DAC and Panobinostat (PAN) co‐encapsulated lysophosphatidic acid receptors (LPAR_1_) targeted lipid nano emulsions (LNEs) could be an alternative therapeutic possibility for triple‐negative breast cancer (TNBCs).^238^ LPAR_1_‐targeted LNEs were fabricated by the addition of 1:1 ratio of lysophosphatidic acid (LPA) to lysophosphatidylcholine (LPC) which is a precursor of LPA. Concisely, LPA and its receptors (LPAR_1–3_) are involved in breast cancer metastasis. G2A is a G protein‐coupled receptor, triggered by lysophosphatidylcholine (LPC), is also overexpressed in human breast cancer. In vitro cell viability study upon TNBC cells (MDA‐MB‐231) exhibited decreased in cell growth after 5 days of treatment with free DAC (12%) and PAN (88%) alone, DAC‐LNEs (30%) and PAN‐LNEs (84%) alone and combination of DAC/PAN (94%) and DAC/PAN‐LNE (92%). Further, DAC/PAN‐LNEs treatment showed increased expression of CDH1(3.8‐fold), an epithelial marker of mesenchymal TNBC, and E‐cadherin (2‐fold) in MDA‐MB‐231 cells due to complete demethylation. Furthermore, expression of oncogene forkhead box M1 (FOXM1) was suppressed (80%) due to the synergistic effect of DAC and PAN when co‐delivered through LNEs.^238^ In addition, Vijayaraghavalu et al. reported the nanogel‐mediated delivery strategy for the cocktail of epigenetic modulators DAC and SAHA which exhibited more synergistic effect as compared to DAC or SAHA alone while pretreated on doxorubicin‐resistant breast cancer cells. IC_50_ values for the combination of both the epigenetic modulators in formulation showed 887 ± 135 versus 3 ± 0.3 which is less than alone treatment of DAC (923 ± 114 vs. 10 ± 0.8) and SAHA (4038 ± 67 vs. 379 ± 4.9) in both solution and nanogel. Moreover, this result manifested the improved efficacy of epigenetic drug cocktail at less concentration when encapsulated in NG.^239^

In another report, research group demonstrated the effect of co‐administration of bacosides encapsulated lactoferrin‐conjugated tri‐block PEG–PLA–PCL‐OH polymersomes (BAN) and coconut oil‐derived Lauric acid containing lactoferrin‐conjugated tri‐block PEG–PLA–PCL‐OH polymersomes (LAN) on scopolamine challenged neurons.^240^ Co‐administration of BAN and LAN unveiled noticeable neuroprotection as well as helped in neurodifferentiation, neurite elongation, and neurogenesis in mouse neuroblastoma and primary neuronal cells. Here, cellular changes were associated with upregulation of BDNF neurotrophic factor and expression of neuronal activity‐dependent Arc gene. Additional investigations revealed that co‐administration of BAN and LAN involved in epigenetic changes by inhibiting DNMTs and HDACs. Overall, this study proved enhanced neuroprotection and neuroenhancement by the coadministration of BAN–LAN than free Rivastigmine and Rivastigmine loaded Lactoferrin‐conjugated tri‐block PEG–PLA–PCL‐OH polymersomes (RiN) due to epigenetic alteration of neuronal genes.^240^

### 6.2 Combination of DNMTi with other therapeutic agents

Over the past few years, several studies have demonstrated the potential benefits of combining DNMTi with chemotherapeutic drugs. This strategy presents a fascinating approach for overcoming the limitations often associated with chemotherapy as a stand‐alone treatment. For instance, combination of conventional treatments with DNMTi has been often explored for the clinical trials for platinum‐resistant or relapsed ovarian cancer as a novel therapeutic strategy. In several Phase I and Phase II clinical trials, researchers have explored the efficacy of integrating decitabine, a DNMTi, with carboplatin, a platinum‐based chemotherapy drug. This combination aims to overcome resistance mechanisms and enhance treatment outcomes in patients facing challenging ovarian cancer situations.^241^ Therefore, a few research groups have delved into the synergy of combining DNMT inhibitors with other chemotherapeutics through a suitable delivery strategy to treat a spectrum of human diseases, spanning from cancers to neurodegenerative conditions which aims to achieve enhanced therapeutic efficacy and superior disease management. Accordingly, a research group reported a combination therapy that contains DNA demethylating agent; DAC and cisplatin‐loaded liposomes as a chemotherapy for triggering the pyroptosis process of solid tumors by caspase‐3 mediation. DAC was treated as preconditioning agent to trigger the pyroptosis during chemotherapy, which may lead to increase the immune response after chemotherapy.^242^ In another research, combination of epigallocatechin‐3‐gallate (EGCG) and ascorbic acid (AA) loaded PEGylated PLGA nanoparticles (EGCG/AA NPs) have been demonstrated for the treatment of AD. Oral administration of EGCG/AA NPs in mice exerted accumulation of EGCG in brain along with other major organs. Further, pharmacokinetic study for oral administration of free EGCG and EGCG/AA NPs revealed similar concentrations of EGCG in initial time, whereas EGCG concentration was found five times higher in long‐term (5–25 h) by EGCG/AA NPs compared to free EGCG. Moreover, EGCG/AA NPs promoted noticeable increase in synapses, as indicated by expression of synaptophysin along with decrease in neuroinflammation with amyloid β (Aβ) plaque burden as well as cortical levels of soluble and insoluble Aβ (1–42) peptide. Thereby, spatial learning and memory has been enhanced significantly.^243^ Furthermore, combination of chemo‐immunotherapeutic approach was also explored by Odunsi et al. on the treatment of epithelial ovarian cancer. Combination of epigenetic modulator DAC and NY‐ESO‐1, a cancer vaccine exhibited disease stability and integrated immunologic responses.^244^

### 6.3 Combination of HDACi with other therapeutic agents

Improving therapeutic outcomes for diseases such as cancer often involves the development of drug combinations, which has emerged as a promising approach. Therefore, targeting numerous pathways involved in cancer frequently leads to synergistic effects and reduces drug resistance. For instance, an FDA‐approved liposome‐based nanomedicine (Vyxeos) containing in a fixed ratio of cytarabine and daunorubicin, has shown significant efficacy in prolonging the persistence of cancer patients.^245^ Compared to stand‐alone drugs, combination therapy through an appropriate carrier often requires lower drug doses to achieve excellent therapeutic effects, resulting in minimizing side effects and toxicity. Therefore, cocktail therapy of HDACis with other therapeutic moieties with an advanced delivery strategies may further enhance the efficacy of cancer treatment as several studies have indicated that HDACs contribute to tumor resistance to chemotherapeutic drugs, and inhibiting HDACs can improve sensitivity towards chemotherapy. Furthermore, HDACis can improve the antitumor immune response through various endogenic mechanisms as current preclinical trials have demonstrated that combining HDACis with other therapeutic agents can produce enhanced therapeutic effects.^246^, ^247^ Consequently, HDACi are widely explored in combination with anticancer drugs including paclitaxel, cisplatin, docetaxel, doxorubicin, gefitinib, erlotinib, and so forth, for the synergistic effects by suitable cargoes. Likewise, Ruttala et al. reported transferrin‐anchored albumin nanocarrier with PEGylated lipid bilayers (Tf‐L‐APVN) for the solid tumor‐targeted co‐delivery of paclitaxel (PTX) and VOR. Cellular apoptosis results demonstrated that treatment with dual drug‐loaded Tf‐L‐APVN showed a significant apoptotic effect on MCF‐7 (~45%), MDA‐MB‐231 (~30%), and HepG2 (~58%) cancer cells while free PTX and VOR did not show any appreciable apoptosis. Moreover, Tf‐L‐APVN‐loaded PTX and VOR exhibited excellent antitumor efficacy by significantly inhibiting the tumor growth in tumor‐bearing mice model.^248^ Another research group fabricated lipid–polymer hybrid nanoparticles (LPH) encapsulating VOR and a microtubule disrupting agent, docetaxel (DTX) (VOR/DTX‐LPH) to attain synergistic anticancer effect.^249^ Further, Kumar et al. demonstrated poly(oligoethylene glycol) monomethacrylate constructed disulfide cross‐linked biodegradable polymeric nanogel (POEOMA) based delivery strategy for VOR and etoposide (ETOP), a topoisomerase II inhibitor. VOR and ETOP‐loaded nanogels exhibited sustained release profile, improved synergistic cytotoxicity, and cell killing efficiency through caspase 3/7 activation compared with free combination of drugs.^250^

In addition, Xu et al. developed supramolecular conjugate of cisplatin (CDDP) and SAHA which was further self‐assembled into nano‐micelles (CDDP‐SAHA‐nano‐micelles) for the synergistic therapy of drug resistance cancer.^251^ Simultaneous release of CDDP and SAHA from nano‐micelles occurs due to hydrolysis of conjugate inside the cancer cells after cellular uptake of CDDP‐SAHA‐nano‐micelles.^251^ Furthermore, SAHA and CDDP‐loaded PEG and PCL‐based hydrogel (SAHA‐DDP/PECE) (PECE = poly (ethylene glycol)‐poly(e‐caprolactone)‐poly(ethylene glycol)) has been investigated in vivo as a controlled release delivery system by another research group for the treatment of oral squamous cell carcinoma (OSCC).^252^ Additionally, POEG‐co‐PVDSAHA (POEG = Poly(oligo(ethylene glycol) methacrylate), PVDF = Polyvinylidene fluoride membrane) based polymeric nanocarrier containing SAHA (POEG‐co‐PVDSAHA) was fabricated by Ma et al., by reversible addition–fragmentation transfer polymerization using the polymer (POEG‐co‐PVD) attached with SAHA through a redox‐responsive disulfide linkage.^253^ Further, POEG‐co‐PVDSAHA was used to formulate tamoxifen (TAM) loaded micelles. In vitro cytotoxicity study against TNBC cell line revealed enhanced and synergistic cytotoxic effect by TAM‐loaded POEG‐co‐PVDSAHA micelles compared with free SAHA and TAM alone and control carrier (TAM‐POEG‐co‐PVMA micelles). Previously, similar research group reported SAHA‐based prodrug polymer (POEG‐b‐PSAHA) using hydrophilic POEG blocks which were further used to formulate DOX‐loaded micelles (DOX/POEG‐b‐PSAHA). DOX/POEG‐b‐PSAHA exerted enhanced therapeutic effect in vivo compared to free DOX and DOX encapsulated POEG‐b‐POM micelles (control).^254^

**FIGURE 7:**
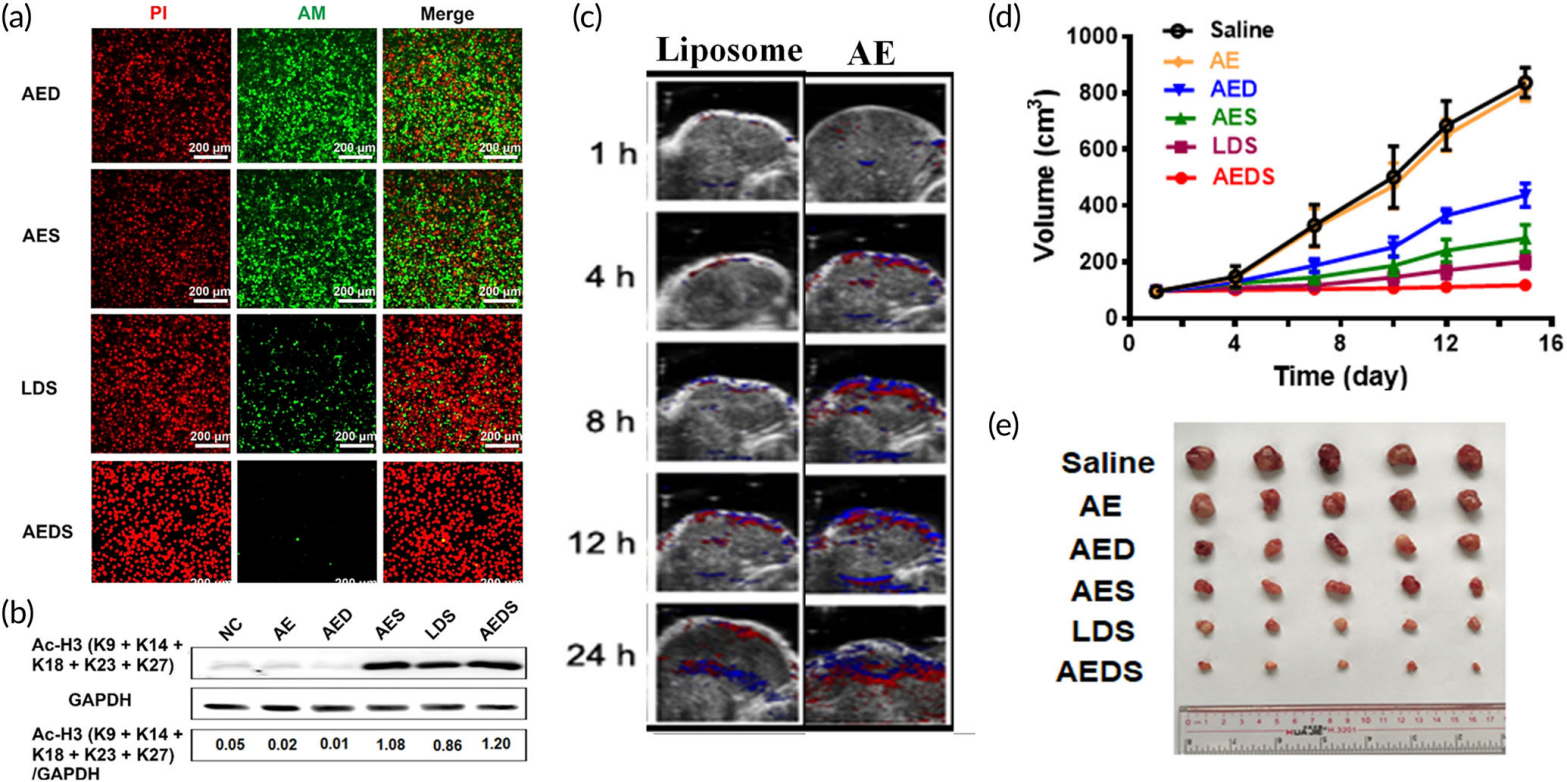
In vitro and in vivo efficacy analysis after treatment with doxorubicin (DOX) and vorinostat (SAHA) loaded artificial exosomes (AEDS). (a) Live‐dead assay result of the inhibitory effects on NCI‐H1299 cells induced by different formulations such as AED (DOX loaded AE), AES (SAHA loaded AE), LDS (dual drug‐loaded liposome), and AEDS. (b) Western Blot analysis of expression of elevated acetylation of histone (K9, K14, K18, K23, and K27) by AES, LDS, and AEDS. (c) The biodistribution analysis by photoacoustic imaging where DiR and Cy5.5 encapsulated AE and liposomes administrated to NCI‐H1299 bearing BALB/C mice. (d) The anti‐tumor efficacy with respect to tumor volume after treatment with AEDS on NCI‐1299 xenografts mice and compared with different groups such as Saline, AED, AES, and LDS. (e) The image of tumor tissues isolated from the different treatment groups such as saline, AE, AED, AES, LDS, and AEDS. Reprinted with permission from Lin et al.^19^

In another study, Lin et al. reported bioengineered nanocarriers such as artificial exosomes (AE) for the combination therapy of DOX and SAHA for the treatment of lung cancer.^19^ These artificial exosomes were developed by thin layer evaporation method followed by decorating the proteins on membrane obtained from NCI‐H1299 cells. Thereafter, DOX and SAHA‐loaded AE were compared with both the drugs loaded conventional liposome with respect to transport efficiency of drugs to the tumor site as well as therapeutic efficacy. In vitro inhibition assay on tumor cells (NCI‐H1299) by dual drug‐loaded AE (AEDS) exhibited significantly improved cell death as compared to single drug loaded AE (AED/AES) and dual drug loaded liposomes (LDS; Figure 7a). Further, western blot analysis revealed that alone AES and AEDS as well as LDS helped to elevate the expression of acetyl histone K9, K14, K18, K23, and K27 significantly (Figure 7b). In vivo biodistribution study in NCI‐H1299 xenografts BALB/C mice model demonstrated the delivery efficiency of AE and liposomes where AE showed faster accumulation as compared to liposome at the tumor site as intense ultrasound occurred from 4 to 24 h of post treatment (Figure 7c). Moreover, AEDS reduced the tumor growth with respect to volume and weight completely whereas, 58% inhibition in tumor growth has been observed for the treatment group of AED and AES (Figure 7d,e).^19^

Another research group synthesized γ‐4‐phenylbutyrate containing a pendant group of ε‐caprolactone monomer (γ‐4‐phenylbutyrate‐ε‐caprolactone) followed by polymerization to form poly (γ‐4‐phenylbutyrate‐ε‐caprolactone) (PPBCL) homopolymer using a catalytic system, NdCl_3_·3TEP/TIBA (TEP = triethyl phosphate, TIBA = triisobutylaluminum). Thereafter, PPBCL has been used for the preparation of DOX encapsulated nanoparticles. DOX loaded PPBCL nanoparticles showed enhanced cell cytotoxicity as compared to free DOX and 20% cell viability upon treating HeLa cells in 24 h while only polymer [PEG‐b‐(PCL‐ran‐PPBCL)] treatment showed similar cell viability in 60 h.^255^ Lee et al. developed curcumin (CUR) loaded 4‐phenylbutyric acid (PBA) conjugated hyaluronic acid (HA) nanoparticles (HA‐PBA/CUR‐NPs) for the treatment of lung cancer. In vitro cytotoxicity assay on A549 cells depicted improved cytotoxic effect by HA‐PBA/CUR‐NPs with less IC50 value (13.0 ± 0.8 μg mL^−1^) as compared to free CUR (18.2 ± 0.1 μg mL^−1^) and combination of free HA‐PBA + CUR (15.9 ± 0.5 μg mL^−1^). Furthermore, in vivo study in A549 cells‐bearing mouse model displayed higher accumulation of HA‐PBA/CUR‐NPs in the tumor site compared with liver and spleen tissue.^256^

Inorganic nanoparticles have also been explored by different research groups for the combination therapy. Accordingly, a research group reported titanium oxide (TiO_2_) based nanoparticles (NPs) incorporating erlotinib (ERL), an EGFR‐TKIs, and VOR for the treatment of breast cancer. ERL‐ and VOR co‐loaded TiO_2_ NPs were able to arrest breast cancer cells (MDA‐MB‐231 and MCF‐7) at G2/M phase leads to show cytotoxic effect. Further, qPCR result indicated upregulation of tumor suppression gene PLAB2 by the treatment of ERL and VOR co‐encapsulated NPs.^257^ Thapa et al. fabricated VOR and bortezomib (BOR) co‐loaded zein nanoparticles (ZNP, ZNP/VB) for treating metastatic prostate cancers with controlled and pH‐dependent drug release profile, enhanced cytotoxicity and apoptosis and increased in vivo antitumor effect as compared to free VOR and BOR alone and their combination.^258^ Another research group reported layer‐by‐layer (LbL) assembled ultrasmall iron oxide (Fe_3_O_4_) based magnetic nanocarriers (MNC) containing the combination of VOR and Tenovir, an anti‐HIV drug for the treatment of neuroAIDS.^259^

In addition to this, Yang et al. fabricated lactoferrin decorated liposome by thin‐film dispersion method for the co‐delivery of Panobinostat (PAN), an HDACi and JQ1, and BRD4 inhibitor for the treatment of colorectal cancer that implied the combination of epigenetic based immunotherapy. This targeted liposomal codelivery mainly focused on lactoferrin‐mediated binding with the LRP‐1 receptor that repolarizing tumor‐associated macrophages (TAM) induced immune checkpoint blockade (ICB) treatment, thus remodeling tumor immune microenvironment.^260^

Besides anticancer treatment, HDACi is also explored with the suitable therapeutic candidates including rosiglitazone, metformin for the treatment of Alzheimer's disease (AD) implicated by brain insulin signal anomalies. A research group reported surface modified with poly(ethylene glycol) (m‐PEG) and poloxamers (PCL) based polymeric nanoformulation (mPEG‐PCL‐NF) containing rosiglitazone and VOR by solvent evaporation method for the treatment of AD. Combination of both the drug‐encapsulated mPEG‐PCL‐NF exerted greater bioavailability with improved therapeutic efficacy alongside improved gene expression for the neurotrophic factors when compared with the combination of free drugs. Moreover, this result indicated the improved synergistic effect of rosiglitazone and VOR due to its delivery strategy.^261^ Similarly, Zheng et al. reported romidepsin and metformin co‐encapsulated poloxamer stabilized polymeric nanocarriers (m‐PEG‐PCL‐NF) for the treatment of AD.^262^

### 6.4 Combination of epigenetic drug and ncRNA

With the advancement in genetic engineering and molecular biology, ncRNAs including siRNAs have gained much more consideration against the treatment of different diseases specifically in cancer, due to their several advantages over small molecules. In contrast, siRNA has limitations such as short circulation time and poor cellular uptake resulting in inadequate clinical applications. To overcome this, co‐delivery of siRNA and small molecules through a suitable carrier is desired to enhance their therapeutic potential.^263^, ^264^ Similarly, Phung et al. created multifunctional nanoparticles co‐encapsulated with VOR and Cellular FLICE‐like inhibitory protein (cFLIP)‐specific small interfering RNA (siRNA) for cancer therapy.^265^ Briefly, mesoporous silica nanoparticles (MSN) containing VOR were formulated by conjugating polyethyleneimine‐biotin (PB) followed by complexation with cFLIP siRNA (VOR/siR@MSN‐PB) electrostatically. Further, the polyethyleneimine (PEI) backbone surface of VOR/siR@MSN‐PB was cross‐linked with a bialdehyde‐modified poly(ethylene glycol) (PEG) through imine linkage (Schiff base; VOR/siR@MSN‐PB‐PEG) resulting in stable and long circulation in blood. VOR/siR@MSN‐PB‐PEG exhibited higher cellular uptake by tumor cells, hence, transcriptional and post‐transcriptional cFLIP expression was inhibited, thereby, the occurrence of cell death via apoptotic signaling pathway. Further, intravenous administration of VOR/siR@MSN‐PB‐PEG NPs in animal models showed high accumulation of VOR/siR@MSN‐PB‐PEG NPs at the tumor site and efficient inhibition efficacy against tumor growth with the safety profiles at low‐dose of VOR.^265^

In another report, Oner et al. demonstrated their research regarding co‐treatment of siRNAs loaded cationic solid lipid nanoparticles (cSLNs) and histone lysine demethylase inhibitor, JIB‐04 (5‐chloro‐N‐[(E)‐[phenyl(pyridin‐2‐yl)methylidene]amino]pyridin‐2‐amine) effective for silencing EphA2 (receptor tyrosine kinase) expression which is involved in developing different cancer including prostate cancer.^16^ Further, trilysinoyl oleylamide (TLO)‐based cationic liposomes (TLOL) have been demonstrated by Shim et al., for the co‐delivery of siRNA and VOR. VOR and siMcl1‐loaded pegylated TLOL complex exhibited decreased tumor growth as compared to free VOR and siGL2‐delivered complex (control), representing synergistic antitumor efficacy by Mcl1‐specific siRNA and VOR when delivered through cationic liposomal system.^266^

### 6.5 Combination of epigenetic drug and other therapies

Phototherapies such as photothermal therapy (PTT), photodynamic therapy (PDT), and radiotherapy along with epigenetic modulators showed promising potential with synergistic effects as well as minimum invasiveness and side effects in the treatment of different human diseases caused by altering epigenetic status. Kaundal et al. reported chemo/photodynamic therapy by casein nanoparticles (CasNPs) prepared by the desolvation method for the treatment of Glioblastoma multiforme (GBM).^267^ More precisely, casein nanoparticles encapsulate an epigenetic modulator genistein (Gen) conjugated with the FDA‐approved fluorophore Indocyanine green (ICG), a hydrophilic tri‐carbocyanine, generally, emits at the near‐infrared range (NIR) between 650 and 850 nm. This photo/chemotherapeutic approach involves ubiquitination‐mediated proteasomal degradation by promoting the depletion of EZH2 and BMI‐1, which activate the production of reactive oxygen species (ROS) alongside apoptosis that authenticates the suppression of PcG proteins. NIR‐induced in vitro study on LN18 glioma cell line, C6 GBM stem cell line, and HEK293T cells has been performed and 350 nM dose of Gen in ICG‐Gen@CasNPs showed more potency as compared to 40 μM IC50 of Gen on LN18 glioma cell line. In addition, more killing efficiency of ICG‐Gen@CasNPs on GBM cells was exhibited with respect to 10% and 20% of cell viability of LN18 and C6 cell lines, respectively with 30 s of NIR exposure. However, no killing was observed in HEK293T cells with and without NIR exposure which confirms the specificity of ICG‐Gen@CasNPs toward GBM cells and GBM stem cells as compared to other cells. Further, trans‐well assay and permeability study along with in vivo and ex vivo biodistribution study revealed promising results that validated the blood brain barrier (BBB) permeation ability by ICG‐Gen@CasNPs.^267^ Sardoiwala et al. investigated that hypericin loaded transferrin nanoparticles (HTfNPs) functioned as a potential targeted photodynamic therapy for the treatment of epigenetic regulated colorectal cancer. Basically, the mechanism involved in PP2A‐mediated BMI1 ubiquitination and proteasomal degradation associated with colorectal cancer; hence, HTfNPs exerted notable therapeutic effects as an anticancer therapeutic to target colorectal cancer. Further, HTfNPs were able to overcome the poor bioavailability as well as compromised hydrophobicity of free drugs.^268^

In another study, Wang et al. investigated that HDACi encapsulated nanoformulation functioned as a potent radio sensitizer for the treatment of solid tumors.^269^ Briefly, PLGA‐lecithin‐PEG core–shell nanoparticles (PLGA‐L‐PEG NPs) have been prepared by modified nanoprecipitaion method where first‐generation HDACi VOR and second‐generation HDACi Quisinostat were encapsulated separately to fabricate two different NPs. Radio‐sensitization experiment revealed that 1 μM concentration of VOR‐NPs, quisinostat‐NPs along with radiotherapy (6 Gy) were more effective on prostate cancer (PC3, DU145), colon cancer (HCT116), and rectal cancer (SW837) cell lines when compared with equivalent dose of free VOR and quisinostat. Moreover, in vivo study revealed drug encapsulated NPs accumulated more in tumor sites than free HDACis and allowed drug release in controlled manner. Hence, this leads to a synergistic effect of HDACi‐NP and radiotherapy.^269^ In addition, Hara et al. reported αPSMA antibodies conjugated gold nanoparticles (pfGNPs) encapsulating Romidepsin/FK228 for the treatment of prostate cancer. In vitro study depicted that pfGNPs showed three‐fold higher accumulation on cells (LNCaP) as compared to nontargeted GNPs. Further, pfGNPs showed cells killing efficiency at 10 μg mL^−1^ concentration from 72 to 24 h based on the dose, whereas FK228 showed cell death efficiency at 1 mg/mL at 24 h. Furthermore, γ‐H2AX assay was determined after radiotherapy (2Gy at 160 kV_p_) and showed significantly increased formation of foci with the treatment of pfGNP (170%) as compared to blank pGNP (158%) and free FK228 (125%). In conclusion, authors demonstrated that antibody‐conjugated FK228 bearing GNPs synergistically enhanced radiosensitization in prostate cancer by improving induction of double‐stranded break in DNA.^270^ Several co‐delivery approaches of epigenetic modulators with other therapeutics and/or other therapies are summarized in Table 3.

**TABLE 3:** Delivery strategies of combination therapy for epigenetic drug with other therapeutics and/or other therapies.

Combination of Drugs/ therapies	Delivery strategy/ system	Applications/Diseases	Mechanism of drugs	Cell line	Outcome	Reference
1. DAC 2. Vorinostat (SAHA/VOR)	Nanogel (NG)	Breast cancer	Inhibition of DNMT and HDAC	MCF‐7/ADR cell	1. Improved efficacy and sustained anti‐proliferative effects than combination of drugs in solution. 2. Both the drugs in combination form in NG were able to achieve IC_90_.	[239]
1. Bacosides 2. Lauric acid (both the drugs are encapsulated in separate formulations)	Lactoferrin‐conjugated tri‐block PEG–PLA–PCL‐OH polymersomes	Neuroprotection and Neuroenhancement for neurodegenerative diseases	Inhibition of DNMT and HDAC	Neuro2a cell	1. Downregulated HDAC5 expression by 0.19‐fold. 2. Significantly reduced expression of DNMT1, DNMT3a, DNMT3b to 0.26‐, 0.16‐, and 0.23‐fold, respectively.	[240]
1. DAC 2. Cisplatin (DDP)	Liposome (DAC was pretreated with DDP containing Liposome)	Triggering the pyroptosis process of solid tumors	Anticancer activity breaking DNA structure with inhibition of DNMT	4T‐1 and CT‐26 cells	1. Improved cytotoxicity with significant increase in pyroptosis process and survival rate of animals. 2. DAC + LipoDDP exhibited enhanced tumor inhibition ability after 2 weeks (average tumor volume < 200 mm^3^) than PBS treated group (tumor volume ~ 1200 mm^3^) in xenografted mice model.	[242]
1. DAC 2. Tumor antigen targeted cancer vaccine: NY‐ESO‐1 3. Doxorubicin (DOX)	Liposome (marketed formulation of DOX‐Liposome was used after chemo‐immuno therapy of DAC+ NY‐ESO‐1)	Human ovarian cancer	Inhibition of DNMT	A2780 and OVCAR3 EOC cell	1. Augmented immune responses with improved antiproliferative activity. 2. Promoter‐specific DNA hypomethylation was observed in patient's blood after treatment.	[244]
1. Etoposide (ETOP) 2. VOR	Nanogel (NG)	Human cervical cancer	Inhibition of DNA topoisomerase II HDAC	HeLa cell	1. Degradation of intracellular glutathione (GSH) was found by NG whereas no degradation was found in extracellular GSH. 2. Increased in vitro cytotoxicity and apoptotic index in tumor cells by VOR/ETOP‐NG.	[250]
1.DDP 2.SAHA	Supramolecular DDP‐SAHA conjugate further self‐assembles into nano‐micelles	Cancer	Anticancer activity with inhibition of HDAC	A549 and A549/DR tumor cells	1. Increased cellular uptake, enhanced cytotoxicity, and greater DNA binding efficacy. 2. Long blood circulation, higher accumulation at the tumor site, with a very negligible systemic toxicity.	[251]
1.Tamoxifen (TAM) 2.SAHA	Micelles	TNBC	Reactivate the expression of functional estrogen receptor α (ERα) with inhibition of HDAC	4T1.2 and MDA‐MB‐231 cells	1. Increased and synergistic cytotoxicity against TNBC cell lines. 2. Significantly enhanced antitumor efficacy in 4T1.2 cell‐induced tumor bearing mice model.	[253]
1.DOX 2.SAHA	Micelles	Cancer	Stabilizing the Topoisomerase II‐DNA complex, and preventing the re‐ligation of DNA strands during replication resulted in double‐stranded DNA break with inhibition of HDAC	4T1.2, MCF‐7, and HCT‐116 cells	1. Sustained release profile till 72 h. 2. More potent cytotoxic effect towards tumor cells. 3. Potential synergistic effect for inhibiting almost tumor growth in vivo with negligible toxicity.	[254]
1. Prodrugs of Butyric acids‐AN7 2. Prodrug of valproic acids AN446 3. DOX	Each prodrug combination with DOX.	Glioblastoma (GBM)	Inhibition of HDAC	U251 MG cells	1. Suppressed the expression of the HDAC1/2 proteins only in the cancer cells. 2. Increased γH2AX (DNA damaging factor) levels by 2.8–3.3 fold by AN446 and AN7 alone and their combination with Dox increased it to 4.7‐fold, whereas Dox alone showed not significant increase in the level of γH2AX.	[271]
1.VOR 2. gefitinib	Hyaluronan‐based nanoparticles	Lung cancer	Inhibition of HDAC and epidermal growth factor receptor–tyrosine kinase inhibitor	H322, H358, and A549 NSCLC cell lines	1. Delayed release pattern of drugs after 5 days. 2. More cytotoxic due to induction of apoptosis as compared to free drugs. 3. Improved inhibition of orthotopic lung tumor growth compared to free drugs in in vivo.	[272]
1.VOR 2. Gefitinib	Trastuzumab decorated mannosylated liposomes (tLGV)	Tumor‐associated macrophage (TAM) reprogramming strategy to overcome EGFR^T790M^ associated drug resistance	Inhibition of HDAC and epidermal growth factor receptor‐tyrosine kinase inhibitor	NSCLC H1975 cells	1. Elevated reactivating oxygen species (ROS) in the cancer cells. 2. Reppressed MsrA leads to the EGFR^T790M^ degradation through 790M oxidation by ROS resulted in resensitizing the EGFR^T790M^‐positive cells to gefitinib.	[273]
1. VOR 2. Bortezomib (BOR)	Zein nanoparticles (ZNP/VB)	Metastatic prostate cancers	Inhibition of HDAC and proteasome which causes the deposition of ubiquitinated proteins resulted in cell cycle arrest and apoptosis of cancer cells	PC3, DU145, and LNCaP cells	1. Higher cytotoxicity and apoptosis due to high uptake in different prostate cancer cells. 2. Increased in vivo antitumor effect with minimal toxicity.	[258]
1. VOR 2. Tenovir	Iron oxide (Fe_3_O_4_) based magnetic nanocarriers (MNC)	NeuroAIDS	Inhibition of HDAC and break the latency in CD4+ T	Primary human brain microvascular endothelial cells and HA cells	1. Sustained drug release (100%) over a period of 5 days. 2. 37.95% ± 1.5% Blood–brain barrier transmigration ability with improved in vitro antiviral efficacy as ~33% reduction of p24 level over a period of 5 days.	[259]
1. Romidepsin 2. Metformin	Poloxomer‐stabilized polymeric nanocarriers (m‐PEG‐PCL‐NF)	AD	Inhibition of HDAC and agonization of PPAR‐γ	–	1. Significantly reduced stress and increased neurotrophic factors resulted in superior neurological effectiveness than the free combination of drugs.	[262]
1. JIB‐04 ‐(5‐chloro‐N‐[(E)‐[phenyl (pyridin‐2‐yl) methylidene] amino] pyridin‐2‐amine) 2. EphA2 siRNA	Cationic solid lipid nanoparticles (cSLNs; siRNA was complexed with cSLNs)	Prostate cancer and tumor spheroids	Inhibition of histone lysine demethylase and silencing of EphA2	PC‐3, DU145 RWPE‐1 and PWR1‐E cells	1. In vitro cytotoxicity was improved during co‐administration. 2. Combinatorial treatment reduced the colony intensity of PC‐3 cells as compared to treatment with siEphA2 alone. 3. Delayed migratory activity of PC‐3 cells as compared to alone treatment with JIB‐04.	[16]
1. VOR 2. siRNA (siMcl1)	Trilysinoyl oleylamide (TLO)‐based cationic liposomes (TLOL)	Cancer	Inhibition of HDAC and silencing of human Mcl1 gene	KB and 293T‐GFP cells	1. Reduction in target protein Mcl1 expression. 2. Significant reduction in tumor growth in animals	[266]
1. Hypericin 2. Photodynamic therapy	Transferrin nanoparticles (HTfNPs)	Colorectal cancer	Activation of PP2A, Caspase3, and inhibition of BMI1, PP2A EZH2, 3Pk, NFκB promotes the ubiquitination/degradation of BMI1	SW480 and HCT116 cells	1. Controlled drug release pattern where 10% release in 16 h, followed by 40% release in 96 h. 2. Two‐fold decrease in IC_50_ as compared to free hypericin. 3. Two‐fold increase in drug half‐time and mean residence time due to long circulation time.	[268]
1. VOR 2. Quisinostat 3. Radiotherapy (VOR and Quisinostat were encapsulated separately)	PLGA‐lecithin‐PEG core–shell nanoparticles (PLGA‐L–PEG NPs)	Solid tumors	Inhibition of HDAC	DU145, PC3, HCT116, and SW620 cells	1. Improved cytotoxic effect as compared to free drugs. 2. Significant increase in foci count after treatment with VOR_NPs/Quisinostat‐NPs followed by radiotherapy as compared to radiotherapy alone. 3. More accumulation in tumor site in in vivo model.	[269]

## 7 MISCELLANEOUS

New research findings demonstrated that epigenetic drugs and their modes of delivery are explored for their therapeutic effects and to check the potency of the epigenetic drugs. A research team led by Lim et al. reported an EPISSAY, a cell‐based assay to check epigenetic drug potency. This EPISSAY system has been developed using a nonmalignant human breast cell line, MCF10A, which expresses silenced triple‐mutated bacterial nitroreductase TMnfsB attached with red‐fluorescent protein (RFP). The EPISSAY has been performed to check the potency of free DAC and DAC‐encapsulated PEGylated liposome, where a 50% greater potency of DAC‐encapsulated PEGylated liposome was observed as compared to free DAC. Further, EPISSAY also determined the potency of HDAC inhibitors, including SAHA. The advantage of this cell‐based bioassay is the novel and rapid system by which efficiency can be determined for the newly fabricated epigenetic drug‐loaded formulations and their existing form of free drug, along with the expression of reactivate genes.^274^

Metformin, a well‐known drug for treating type 2 diabetes, has been used for PD and colorectal cancers by Sardoiwala et al. and Abd‐Rabou et al.^192^, ^275^ Abd‐Rabou et al. prepared metformin‐loaded lecithin nanoparticles for treating colorectal cancer mediated by modulating noncoding RNAs epigenetic based pathway.^275^ Apart from this, nanostructure constructing materials such as poly(amidoamine) (PAMAM) which is majorly involved in forming dendrimers, are reported for their potential activity against epigenetic alteration. Kohonen et al. reported that PAMAM exhibited regulation of DNA methylation and histone modification even at a micromolecular level without any significant changes in cell viability.^276^ Besides, xenon, a noble gas, has also been explored as a xenon‐loaded liposome for treating long‐term neuroprotection after stroke through an epigenetic‐mediated pathway by Huang et al.^277^ In addition, epigenetic modulator is also involved for preparing “fusion drug” with the support of another drug. Likewise, Liao et al. reported RHA‐loaded nanoparticles for melanoma therapy where RHA is a fusion drug of well‐known HDACi, VOR, and all trans‐retinoic acid (ATRA). However, in vitro study depicted that only 1% of HDAC inhibition activity was found by RHA as compared to VOR.^278^

## 8 MAJOR CHALLENGES ASSOCIATED WITH EPIGENETIC DRUGS AND THEIR DELIVERY STRATEGIES

So far, we discussed the epigenetic therapeutics and their associated delivery methods. These delivery strategies encompass a range of carriers, which have been discussed in the preceding section (Section 4). These carriers include nanoparticles (NPs), liposomes, dendrimers, and nanogels, as well as prodrugs and drug combinations. The development of both epigenetic drugs and their delivery systems is still in the early stages as there is limited research on their application. Although many epigenetic targets and enzymes exist, here we have highlighted only those for which drugs and delivery strategies have been developed. Ongoing research aims to identify additional targets and design/synthesize new inhibitors/activators and/or modify existing ones. This section addresses several challenges related to epigenetic modulators which may present challenges during their delivery and subsequent translation into clinical settings.

Recognizing the appropriate target is essential for developing new therapeutic strategies for any disease, and this is equally important in the field of epigenetics. The challenge in selecting an appropriate epigenetic target comes from the large number of closely interacting targets and the complexity of their interactions with other proteins and nucleic acids. Determining the best intervention points in cellular activity is difficult because the epigenome is inherently more dynamic than the genome.^279^ Human histones serve as a case study illustrating the complex interactions between ubiquitination and deubiquitination cycles. These cycles involve the addition and removal of monoubiquitin molecules, which signal to other epigenetic modifiers and gene regulatory mediators.^280^ Despite identifying targets related to epigenetics for specific diseases, several challenges must be overcome to successfully design epigenetic drugs. These challenges involve understanding the complexities of epigenetic enzymes functioning within multimeric complexes, confirming affinity for specific enzyme isoforms, synthesizing dual inhibitors, distinguishing between histone and non‐histone substrates, and optimizing different combinatorial therapies for epigenetic drugs. Additionally, developing small molecules for epigenetic targets presents several challenges. Many of these targets belong to enzyme classes that are understated by existing therapeutic molecules.^281^ However, a substantial portion of the chemical landscape/domain remains available for exploration. In addition, most epigenetic modulators exhibit stability issues, poor pharmacokinetic profiles, and challenges related to biodistribution. HDACi has achieved only inadequate success due to various factors that include poor pharmacokinetics, low solubility, short half‐life, rapid metabolism, and clearance, lack of tissue/cell permeability, as well as the development of drug resistance.^282^ Similar issues have also been observed with DNMTi, such as DAC and AZA.^87^ Targeted distribution and controlled drug release may improve the effectiveness of different epigenetic modulators. Additionally, the biodistribution pattern of epigenetic modulators needs significant enhancement due to their poor pharmacokinetic profiles. Besides, there is a requirement to create metabolically stable drugs, ensuring they remain in the bloodstream for prolonged periods to demonstrate potent activity. As discussed in various reports, nanomedicines can enhance effectiveness and minimize side effects by optimizing pharmacokinetics, and facilitating targeted drug delivery to specific sites. Biodistribution assessments of nanomaterials are crucial for refining their biological applications and understanding their distribution in various organs. Further, the observed cytotoxicity in clinical studies may come from the disruption of multiple pathways by these inhibitors and nonspecificity towards target enzymes. Sometimes inhibiting HDACs can affect the CpG methylation of certain gene promoters by altering the activity of DNMTs.^283^ Common side effects reported with HDAC inhibitors include thrombocytopenia, neutropenia, anemia, fatigue, and diarrhea. In certain cases, discontinuing the medication promptly reverses HDAC‐induced thrombocytopenia.^284^

Researchers are actively exploring various delivery strategies to address the challenges associated with several difficult‐to‐deliver molecules including epigenetic drugs. Once these modulators are refined with respect to pharmacokinetics, specificity, and safety, optimizing delivery strategies becomes crucial for enhancing their efficacy and further minimizing adverse effects. This sequential approach underscores the need for comprehensive solutions in both drug development and delivery to realize the therapeutic potential of epigenetic interventions.

## 9 CONCLUSION AND FUTURE ASPECTS

Research groups are synthesizing new epigenetic modulators, making prodrugs of existing epigenetic modulators to find their efficacy in altering epigenetic status and overcoming the foretold research gap and disadvantages of the existing drugs. Conversely, researchers are also exploring the repurposed therapeutic potential for epigenome editing of a few drugs used, which are well‐known and documented previously for treating specific diseases. On the other hand, a few combinational therapies of epigenetic drugs have also been exploited for improved and sustainable therapeutic effects. Although several studies have been reported on the delivery strategy of epigenetic modulators to prove their efficacy, most of the studies were demonstrated in vitro. So, extensive in vivo studies such as gene expression governed by the epigenetic mechanism, in vivo drug formulation stability, and the pharmacokinetic and in vivo biodistribution of the epigenetic drug‐loaded formulation need to be performed to confirm the results that are in accordance with the in vitro studies. Surface‐modified delivery strategies could be an exciting area for epigenetic research as retention and targeting properties will be enhanced by avoiding toxicity with an improved therapeutic index. As discussed above, delivery strategies for epigenetic modulators, including HATi, HMTi, Histone ubiquitination, phosphorylation, and SUMOylation, are not extensively explored. Further, delivery strategies for epigenetic modulators conjugated with nucleic acid‐based bio‐molecules such as ncRNA and siRNAs. miRNAs and CRISPR‐Cas‐based systems are in a naive stage in design and assessment. Many reports suggested that these molecules showed promising effectiveness and reduced off‐target effects. Interestingly, there are boundless opportunities to explore the therapeutic properties of delivering more than one biomacromolecule, including CRISPR‐Cas‐based EEs, to treat or regulate various diseases through epigenome editing.

## AUTHOR CONTRIBUTIONS

**Sonia Guha:** Conceptualization; writing – original draft; writing – review and editing. **Yogeswaran Jagadeesan:** Writing – review and editing. **Murali Monohar Pandey:** Writing – review and editing. **Anupama Mittal:** Writing – review and editing. **Deepak Chitkara:** Conceptualization; writing – review and editing; supervision; funding acquisition.

## CONFLICT OF INTEREST STATEMENT

The authors declare no conflict of interest.

## Data Availability

Data sharing is not applicable to this article as no new data were created or analyzed in this study.
